# Convective storms alter bioaerosol populations through cold pools and precipitation

**DOI:** 10.1039/d5ea00129c

**Published:** 2026-01-06

**Authors:** Teresa K. Feldman, Chamari B. A. Mampage, Nicholas M. Falk, Janeshta C. Fernando, Brian Heffernan, Thomas C. J. Hill, Drew Juergensen, Claudia Mignani, Marina Nieto-Caballero, Leah D. Grant, Susan C. van den Heever, Paul J. DeMott, Sonia M. Kreidenweis, Russell J. Perkins, Elizabeth A. Stone

**Affiliations:** a Department of Chemistry, University of Iowa Iowa City IA 52242 USA betsy-stone@uiowa.edu; b Department of Atmospheric Science, Colorado State University Fort Collins CO 80526 USA russell.perkins@colostate.edu; c Water and Soil Resource Research, Institute of Geography, University of Augsburg 86159 Augsburg Germany; d Department of Chemical and Biochemical Engineering, University of Iowa Iowa City IA 52242 USA

## Abstract

Meteorology can alter bioaerosol properties, potentially enhancing their impact on public health and cloud microphysics. The BioAerosols and Convective Storms (BACS) study was conducted over May–June 2022 and 2023 in Northern Colorado and examines how convective storm processes such as precipitation and cold pools affect bioaerosol concentrations and properties, including pollen, fungal spores and bacterial endotoxin. The two seasons were vastly different climatologically, with drought-like conditions and greater endotoxin concentrations during 2022 and near record rainfall with higher fungal spore concentrations during 2023. Online (fluorescence) and offline (chemical tracer) measurements were used to characterize bioaerosols, alongside collocated measurements of ice-nucleating particles (INPs). Precipitation events generally increased supermicron fluorescent particle concentrations which consisted primarily of fungal spores, as determined from fungal spore counts, chemical tracers, and fluorescent particle types. Storm-generated cold pools had more varied impacts on bioaerosols, sometimes causing depletion and other times enrichment, with peak fluorescent particle concentrations correlating significantly with cold pool strength (*r*_s_ = 0.79, *p* < 0.05, *n* = 12), indicating that stronger cold pools produce greater increases in local bioaerosol concentrations. Biological INP concentrations in air active at warmer than −15 °C from 1–10 µm in size were enhanced by roughly one order of magnitude in samples collected during convective storms compared to pre-rain samples. Contributions of fungal spores to the enhanced INPs were supported by a significant correlation between large (2.5–10 µm) heat-labile INP concentrations active at −15 °C with mannitol, a fungal spore tracer (*r* = 0.91, *n* = 8, *p* < 0.01). This study found convective storms can greatly increase boundary-layer concentrations of fungal spores and warm-temperature biological INPs, leading to high exposure risks for sensitized populations and the potential for bioaerosols to influence cloud processes.

## Abstract 2

Thunderstorms and precipitation can alter bioaerosol properties and concentrations, potentially enhancing their impacts on public health and cloud microphysics.

## Graphical abstract

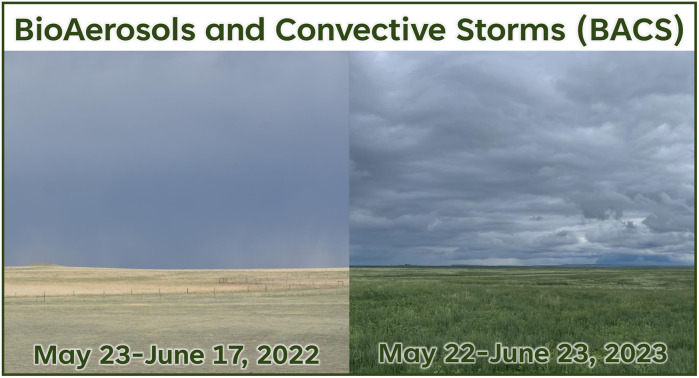

**Environmental significance**

Bioaerosols can affect the Earth's climate by impacting cloud microphysics, particularly by serving as ice-nucleating particles (INPs). The current study examines how convective storm processes such as precipitation and cold pools affect bioaerosol concentrations and properties. We present evidence of significant increases in bioaerosols and biological INPs during convective storms that we primarily attributed to fungal spores. Rainfall significantly enhanced bioaerosol concentrations above pre-storm conditions, while storm-generated cold pools had more varied impacts on bioaerosols. Stronger cold pools with greater perturbations of temperature, wind speed, and humidity generally led to greater bioaerosol enhancements. Our results demonstrate convective storms can greatly increase boundary layer fungal spore and biological INP concentrations through cold pools and precipitation.

## 1 Introduction

Primary biological aerosol particles (hereafter “bioaerosols”) can have significant impacts on human health and the climate due to their inherent properties and sizes. Common bioaerosols include pollen (10–100 µm), fungal spores (1–50 µm), and bacteria (0.25–8 µm), with typical number concentrations during the growing season of 10^1^–10^3^ grains m^−3^, 10^3^–10^4^ spores m^−3^, and 10^4^ bacteria m^−3^ respectively.^1–3^ Bioaerosols can be allergenic, toxic, and/or pathogenic, triggering negative health impacts.^4^ Bioaerosols can also impact clouds by acting as cloud condensation nuclei (CCN)^5,6^ and ice-nucleating particles (INPs)^7–9^ that influence microphysical, dynamical and precipitation processes, with subsequent implications for climate. As INPs, bioaerosols are effective at seeding ice for warm sub-freezing temperatures (>−15 °C),^10,11^ which are important to consider for cloud development through triggering secondary ice production processes.^12–14^ The effect of bioaerosols on human health and on weather and climate can be altered by atmospheric conditions and cloud processes, through changes to the sizes, concentrations, and types of bioaerosols present.^1,2,15–18^ Because of the complex nature of bioaerosol impacts and their importance to human health and climate, the current study examines how convective storms alter the properties of bioaerosols.

Convective storms can potentially impact the transport and surface-atmosphere exchange of bioaerosols through features like updrafts, downdrafts, precipitation, and cold pools. While precipitation can act as a particle sink, it can also increase concentrations of bioaerosols^15,19,20^ through mechanisms of surface rain splashing,^21–25^ active surface emission of fungal spores,^26^ and pollen rupture due to high relative humidity, at the surface or aloft, releasing pollen fragments.^16,27^ Bioaerosols have been implicated as triggers for thunderstorm asthma epidemics due to pollen rupture in the storm^17,27^ and increased fungal spore counts,^28,29^ which can exacerbate respiratory conditions, especially allergic asthma and rhinitis in sensitized individuals.^29–31^ Convective storms increase exposure risk for sensitized individuals, hence their roles in enhancing bioaerosol concentrations in air are important to understand to protect populations sensitive to aeroallergens.

Cold pools are negatively buoyant regions of air created through the evaporation and/or melting of the precipitation produced by convective storms. This cooled air is denser than its surroundings, causing it to sink and spread laterally upon reaching the surface.^32,33^ Cold pools with greater temperature perturbations from their environments are stronger and travel faster than those with weaker perturbations. Reported temperature deficits of midlatitude continental cold pools can be as large as 18 °C, with stronger cold pools typically having temperature deficits of more than 4–5 °C.^34–38^ The leading edge of a cold pool is termed a gust front, typically characterized by enhanced wind speeds and a change in wind direction due to the lateral movement of the cool, dense air displacing the warmer air ahead of it. Cold pools are effective at mechanically lofting particles, thereby altering near-surface layer particle concentrations.^39,40^ A study by Seigel and van den Heever^40^ demonstrated that cold pools can loft dust and lead to dust ingestion within the storm updraft itself, with the quantity dependent on the pre-storm environment and the nature of the storm. Similarly to dust, bioaerosols, if lofted by cold pools, have the potential to impact the storm structure itself by altering the cloud microphysics and precipitation processes.^41–43^

Bioaerosols are often efficient in activating freezing at temperatures warmer than −20 °C, which we will hereafter refer to as warm-temperature INPs. These warm-temperature INPs are expected to be important for cloud and precipitation development, with ambient concentrations that can be sensitive to environmental conditions. Several studies have found that biological particles are the dominant contributors to warm-temperature INPs,^41,44–46^ indicating the potential of bioaerosols to alter local hydrological cycles. Across common bioaerosol types, INPs have been identified to be sourced from species of bacteria (*e.g. Pseudomonas syringae*),^11,47–49^ fungi including spores (*e.g. Cladosporium herbarum*),^15,50–54^ pollen (*e.g. Betula alba*),^55–57^ and pollen fragments (*e.g. Betula pendula*).^58^ Precipitation has been demonstrated to increase local biological INP concentrations,^15,25,59–61^ while one study showed that cold pools can effectively increase INP concentrations but have not been evaluated for their impact on biological INPs specifically.^62^ To fully assess the potential impact of INPs on clouds through ingestion by convective storms, characterization of changes to biological INPs during storms is critical.

Traditional bioaerosol measurements have relied on offline methods, including chemical and biological tracer analysis, with more recent measurements utilizing online methods for higher time resolution. Sucrose and fructose have previously been used as markers for pollen due to their large mass contributions to the pollen grain as energy storage materials.^20,63–66^ Mannitol has been utilized as a tracer for fungal spores, as it stores energy in the spore and correlates with ambient fungal spore counts.^67,68^ Endotoxins are lipopolysaccharides that are present on the cell walls of Gram-negative bacteria, which are bacteria with an inner and outer membrane in their cell envelope. Endotoxin is an inflammatory agent^4^ and has been correlated with Gram-negative bacteria.^69,70^ Online methods include the Wideband Integrated Bioaerosol Sensor (WIBS), an online single particle fluorescence spectrometer, which characterizes fluorescent particles as a proxy for bioaerosols. Bioaerosols are targeted by utilizing wide fluorescence emission bands that encompass common biological fluorophores.^71^ Non-biological fluorophores can interfere with WIBS signals, including species like soot, humic-like substances, secondary organic aerosols, and mineral dust, but the extent of this interference is expected to be weak under remote conditions and to primarily contribute to particle types not strongly associated with biological material.^71–75^ Pollen, fungal spores, and bacteria exhibit characteristic particle sizes and fluorescent particle types in WIBS,^76–78^ although WIBS alone cannot provide bioaerosol type identification. Combining offline and online measurements allows for the chemical tracer measurements to support assignment of bioaerosol types to the highly time resolved measurements from the WIBS.

Understanding the impacts of storm events on bioaerosol populations is crucial for understanding and ultimately predicting their impacts on health and climate. In this study, we expand upon prior knowledge by leveraging fluorescent particle measurements with chemical tracers and microscopy to understand how precipitation and cold pools associated with convective storms alter bioaerosols and biological INPs in a semi-arid grassland environment in Northern Colorado. To assess how convective storms impact bioaerosols, the primary objectives of this study were to characterize how rain and cold pools change the (1) concentrations, (2) sizes, and (3) types of bioaerosols present, and (4) understand the contributions of bioaerosols to local INP concentrations. Specifically, precipitation and cold pools were targeted for their potential to change the surface-atmosphere exchange of biological particles during storms. This work is a part of the BioAerosols and Convective Storms (BACS) study,^79^ focused on characterizing potential feedbacks between convective storms and bioaerosols, with collaborative research focused on understanding storm processes and aerosol characteristics. Through a combination of chemical tracer analysis, particle fluorescence, and INP analysis, this study provides characterization of convective storm impacts on bioaerosols in the grasslands region of the west-central United States.

## 2 Methodology

### 2.1 Field site description

Field research for the BACS study was conducted from May 23 to June 17 of 2022 (BACS-I) and May 22 to June 23 of 2023 (BACS-II), at the Central Plains Experimental Range (CPER), which is a National Science Foundation National Ecological Observation Network (NEON) site located in Nunn, Colorado, USA (40.8155, −104.7456). The CPER site is a semi-arid grassland with primary vegetation of shortgrass steppe, and an average elevation of 1654 meters (Field Sites: Central Plains Experimental Range NEON, 2025). CPER was chosen as it is a remote environment, with limited anthropogenic influence, and provides access to a NEON flux tower equipped with a sonic anemometer and gas sensors. Additionally, this site is situated within a grassland environment that experiences significant weather changes, including severe storms and their associated hazards such as tornadoes and flash flooding.^38,80^ The location for ground-based sampling and tower measurements is used for grazing cows that were present during the period of fieldwork. A gravel road is located approximately 400 meters south of these sites. Neither local vehicle traffic nor foot traffic were detected in fluorescent particle (FP) measurements during recorded site visit times. The Semi-arid Grassland Research Center (SGRC), which was used for substrate changes, was located roughly 2.7 km away from the sampling site (40.8122, −104.7773).

### 2.2 Online measurements

Meteorological data for BACS-I were retrieved from sensors located on the flux tower and in the soil plot for wind speed and direction,^81^ barometric pressure,^82^ and temperature and relative humidity.^83^ Due to issues with the NEON precipitation measurements, precipitation data was obtained from the National Atmospheric Deposition Program site (CO22; 40.806, −104.756)^84^ which is located ∼1.4 km southwest of the NEON tower, resulting in more uncertainty in rainfall timing at the soil plot for BACS-I. For BACS-II, a meteorological station (Davis Instruments, Vantage Vue) attached to the high flow impactor (HFI) housing at a height of 2.6 m above ground level (AGL) was used to obtain meteorological data, except for pressure, which was retrieved from NEON due to measurement issues. Thermodynamic perturbations of cold pools (CPs) were found by taking the difference between the peak value (local maxima or minima) found during the CP passage and a ten-minute averaged background. The CP start was determined based on the beginning of the temperature decrease and wind speed increase. The CP duration was determined by temperature and wind speed stabilizing and/or returning to pre-CP levels. Virtual potential temperature (*θ*_v_) was calculated for both campaigns using meteorological data from the meteorological station and NEON tower.

The Colorado State University Continuous Flow Diffusion Chamber (CFDC) allows for continuous measurements of INPs to observe their temporal development. The instrument is an ice-thermal gradient diffusion chamber that measures INPs by freezing aerosols and optically detecting frozen particles. The theory of operation behind the instrument is described by Rogers,^85^ Rogers *et al.*,^86^ and Eidhammer *et al.*,^87^ with the “HIAPER” CFDC configuration used for BACS described by DeMott *et al.*^88^ Additionally, an aerosol concentrator was utilized to concentrate coarse mode particles in the ∼250 L min^−1^ inlet flow into 1.5 L min^−1^ exit flow that was sent to the CFDC,^89,90^ with a concentration factor (CF) used to scale the INP measurements. The CF was determined from the ratio of INPs with and without the aerosol concentrator and estimated at 20-fold for periods when ambient INP concentrations were below the CFDC limit of detection, preventing calculation of CF. This estimation is based on CF values obtained for other time periods, and since temporal evolution of INPs is primarily examined using the CFDC data in this work, accurate calculation of CF is not critical. In order to prevent false detections of large hygroscopic aerosol as INPs in the CFDC, a two-stage 2.5 µm impactor was utilized in between the aerosol concentrator and the CFDC. This does limit the instrument's ability to detect some materials, such as large intact fungal spores or pollen grains, so changes in these materials, but importantly not their fragments, will not be captured in INP timeseries. During operation, temperatures in the CFDC were typically around −20 °C, with supersaturations of approximately 5%. This puts measurements in the immersion nucleation mode, where all aerosol particles within the instrument will activate as liquid droplets and those containing ice nucleating entities active at the measurement temperature will freeze and be detected as INPs. Details on operation of the CFDC and data processing can be found in the read me file for the associated data archive at the National Center for Atmospheric Research Earth Observatory Laboratory data archive.^91^ CFDC concentrations here are reported per standard volume with reference conditions of 25 °C and 101.325 kPa.

A Wideband Integrated Bioaerosol Sensor (WIBS) – 5 (Droplet Measurement Technology), a single particle fluorescence spectrometer, was affixed to the flux tower at a height of approximately 9 m AGL to provide high-time resolution measurements of bioaerosols (maximum time resolution of >20 Hz). Air was pumped to the instrument through a total suspended particulate inlet (Mesa Laboratories, MiniPM TSP Sampling Head) at a flow rate of 5 L min^−1^. The sampling head and connective tubing used were chosen to minimize loss of larger particles (>10 µm), with sampling efficiency for particles dropping off from roughly 90% to 50% for particle sizes of 10 µm to 20 µm, respectively. The WIBS uses fluorescent particles as a proxy for biological particles in the atmosphere as most biogenic particles have intrinsic fluorescence due to known biological fluorophores.^71^ A WIBS-4a instrument was also operated at the SGRC roughly 1.5 m AGL during the BACS-I and BACS-II campaigns. The WIBS-5 measures particles ranging in size from 0.5–30 µm with a sample flow rate of 0.3 L min^−1^ and a sheath flow rate of 2.1 L min^−1^. The WIBS-4a operates with the same sample and sheath flow rates, but only measures particles ranging in size from 0.8–15 µm. Sizing by the WIBS-5 and WIBS-4a is calibrated to polystyrene latex (PSL) microspheres (refractive index of 1.58), and particle sizes are calculated from scattering of a 635 nm diode laser using Mie theory (WIBS-NEO Operator Manual).

The WIBS excites particles sequentially with two Xe lamps at 280 nm and 370 nm, and fluorescence emission is detected by two photomultiplier tubes (PMTs) in the ranges of 310–400 nm and 420–600 nm, resulting in three fluorescence channels described by excitation at 280 nm with emission between 310–400 nm (A), excitation at 280 nm with emission between 420–600 nm (B), and excitation at 370 nm with emission between 420–600 nm (C). These excitation-emission wavelength ranges capture fluorescence by tryptophan, riboflavin, and NAD(P)H, as well as other biogenic fluorophores.^71^ A particle can fluoresce in one or multiple channels (A, B, and C), which produces a total of seven types of particle fluorescence that can be characterized using the following notation: A, B, C, AB, AC, BC, and ABC.^92^ To define particles as fluorescent, fluorescent thresholds for the WIBS-5 and the WIBS-4a were set for each channel based on an average forced trigger, recorded fluorescence when the chamber was empty, plus three times the standard deviations. To establish background signals and fluorescence thresholds, empty chamber fluorescence measurements were taken for ten seconds every eight hours and were averaged over four to seven days. To minimize potential influences from non-biological fluorescent species, B- and BC-type particles alone were not used in assigning biological particle types to FP responses. Particle concentrations in the WIBS data were averaged over one-minute intervals. Campaign average concentrations and fractions were calculated for BACS-I from May 22, 2022 21:57–June 17, 2022 23:59, and for BACS-II from May 23, 2023 10:06–June 23, 2023 23:58. All data analysis for the WIBS-5 was completed in IgorPro using the WIBS toolkit provided by Droplet Measurement Technology for the WIBS-5.

### 2.3 Offline measurements

Samples were collected for offline measurements of rainwater, pollen counts, and aerosol composition and properties. Instruments used for offline measurements were installed in soil plot 3, located roughly 150 meters NW of the NEON flux tower (40.8163, −104.7464). Rainwater was collected throughout the campaign with a single chimney atmospheric precipitation sampler (N-CON, 125 GS), at a collection height of 1.2 m AGL. All Pyrex glassware used in collection was initially cleaned with ultrapure water with a resistivity of 18.2 MΩ cm^−1^ (Barnstead EasyPure II, 7401) and baked at 450 °C for 5 hours, while subsequent cleanings included 10 washes with deionized (DI) water after rainwater collection in the field. Corning centrifuge tubes (50 mL) were affixed to the side of the chimney to collect water for offline INP analysis. Water collected in the glass sample bottle, through a conical glass funnel with a diameter of 11.68 cm, was transferred to centrifuge tubes for storage.

A Burkard spore trap (Burkard Manufacturing Company) with a 2 mm inlet was operated at 2.0 m above the surface with a flow rate of 10 L min^−1^ to collect pollen and fungal spores. The Burkard operates with an internal gear that moves the slide at a rate of 2 mm h^−1^, which allows for hourly time resolution of pollen and fungal spore concentrations. Substrates for collection were 75 × 25 × 1.0 mm glass microscope slides (VWR, 48300-026) coated with a layer of Lubriseal grease (Thomas Scientific). The slides were stored at room temperature in a desiccator following collection.

A sequential aerosol sampler was fabricated for this study to collect atmospheric particle samples for dry and wet conditions. Two high-flow cascade impactors (HFIs, TSI, model 129) along with their vacuum pumps (TSI, model 0130-01-1051) were stored in housing made up of two vented electrical boxes stacked on top of each other. The impactors operated with a flow rate of 100 L min^−1^ which was measured daily with a Rotameter (Clarkson, GF-6540-1250) after the samples were collected and before starting a new sample period. The average flow rate was then used to calculate the volume of air for sample periods. Each impactor was connected to a total suspended particle (TSP) inlet (Tisch, TE-TSP-D) 2.0 m and 2.1 m above the ground for BACS-I and -II, respectively. The HFI pumps were connected to a precipitation monitor (Thies CLIMA, 5.4103.10.000) located at a height of 1.8 m AGL to collect samples before, and then during and after rain events. Samples beginning with the start of rain were stopped and collected the following morning.

Cascade impactors provide size resolved samples on the basis of larger particles having more inertia, resulting in their impaction onto the substrate, while the smaller particles can follow the path of air to the next stage. The HFIs had a pre-filter, four stages, and an after-filter, with 50% cut-off aerodynamic diameters of: 25 µm, 10 µm, 2.5 µm, 1 µm, and 0.25 µm. For the pre-filter and four stages, 76 mm polycarbonate substrates with 0.05 µm pores (Whatman, 111503), cleaned with 5% hydrogen peroxide and DI water, were used. A 90 mm quartz fiber filter (Pallflex, 7203) was used as the after-filter, and was baked at 550 °C for 18 hours. All samples were handled in accordance with clean-handling techniques developed for the measurement of INPs.^93^

### 2.4 Sample collection, storage, and processing

Samples for ground-based offline instruments were retrieved daily, with filter changes occurring in the morning between 08:00–09:00 local time. Impactor samples were changed inside a laminar flow hood during BACS-I and in a clean room with two HEPA air filters during BACS-II. Field blanks were collected once every five samples by placing the substrates in the impactor and pulling no air through. Collected polycarbonate filters were placed in aluminum foil envelopes using plastic forceps (Fine Science Tools, 11700-01) which were cleaned with DI water and Windex, while the quartz fiber filters were placed into 90 mm Petri dishes with baked aluminum foil cups (BACS-I) or in foil envelopes (BACS-II). Following collection, all rain and HFI samples were stored at −20 °C. Sonication in DI water for 10–15 seconds was used to clean the impactor stages (roughly once a week) and impaction plates (once a day). Samples collected during the BACS-I campaign were subsampled into four quarters in a laminar flow hood using a foil guide, with handling techniques referenced previously, and a ceramic knife (Kyocera, FK-075WH). Blades were cleaned every five uses with Windex and DI water and cleaned with Clorox bleach spray following every use. During BACS-II, most subsampling occurred during the field campaign in the clean room during sample changing.

### 2.5 Sample analysis

#### 2.5.1 Pollen analysis

The Burkard slides were mounted and imaged to find pollen counts throughout the sampling periods. Slides were mounted with coverslips using a solution containing trace amounts of basic fuchsin stain and phenol, glycerin (35 mL), gelatin (5 g), and water (35 mL), and the edges were coated with sealant (Cover Grip, Biotium). The samples were imaged at the University of Iowa Central Microscopy Research Facility using brightfield light microscopy (Olympus BX-61) at a magnification of 10× for pollen counts. To find average hourly concentrations, the longitudinal counting method was used for two 1 × 48 mm transects, which is outlined by Hughes *et al.*^16^ in their SI. For each transect, pollen was counted in 2 mm sections which correspond to one-hour increments, with the average count between transects used to calculate hourly concentrations. Identification of pollen and spore types was performed for periods of interest at a magnification between 10–40× based primarily on work by E. G. Smith.^94^

#### 2.5.2 INP analysis

One fourth of each HFI filter was analyzed for INPs using Colorado State University's Ice Spectrometer (IS). A detailed description of the IS in a general form can be found in Hiranuma *et al.*^95^ and DeMott *et al.*,^96^ with a summary provided here. Samples were placed in sterile 50 mL polypropylene tubes (Falcon), 8 mL of 0.1 µm filtered deionized water was added, and tubes were then tumbled end over end (60 cycles min^−1^, 20 min) to suspend particles. Heat treatment of suspensions prior to analysis was performed for a subsection of samples to degrade proteins in biological INPs, with suspensions exposed to 95 °C for 21 minutes. Peroxide treatments were also carried out for a subset of samples to degrade most types of organic materials, with immersion in 10% hydrogen peroxide for 21 min followed by neutralization of peroxide using catalase. These treatments are described in detail in Tobo *et al.*^97^ and McCluskey *et al.*^98^ For analysis, aliquots of aerosol suspensions are added to sterile wells, the temperatures are then lowered down to −30 °C (−0.33 °C min^−1^) and frozen wells are counted as temperatures decrease (≤0.5 °C intervals). Cumulative concentrations of INPs are estimated based on equations from Vali,^99^ as with error estimations, including field blank subtractions, described by Creamean *et al.*^100^ Differences between untreated and heat/peroxide treated samples were used to determine populations of INPs which were likely biological (heat-labile) and organic (degraded by peroxide) in nature.

#### 2.5.3 Carbohydrate analysis

The samples collected with the sequential aerosol sampler were extracted and analyzed for carbohydrate tracers of pollen and fungal spores. All glassware used in analysis was rinsed 5× each sequentially with tap, DI, and ultrapure water (18.2 MΩ cm^−1^), before being baked at 500 °C for 5.5 hours. Sample vials, centrifuge tubes, and plastic syringes were rinsed 5× with ultrapure water (18.2 MΩ cm^−1^) and allowed to dry prior to use. Samples collected with the HFIs were analyzed for carbohydrates following the method described in Rathnayake *et al.*^20^ A quarter of each sample from the four lowest stages and the after filter of the impactors was extracted into 4.00 mL of ultrapure water through 10 minutes of rotary shaking at 125 rpm (VWR, Advanced Digital Shaker), followed by 30 minutes of sonication at 60 Hz (Branson, 5510), and then shaken again for 10 minutes at the same speed before being passed through polypropylene syringe filters (0.45 µm). For analysis, 0.5 mL of extract were run on the instrument, and the remaining extract was stored at −20 °C.

Samples were analyzed using high performance anion exchange chromatography with pulsed amperometric detection (HPAEC-PAD). Extracts were run on an ICS-5000 (Dionex) system using Chromeleon 7 software for data analysis, with a CarboPac PA20 analytical column and amino trap guard column. Pulsed amperometry was used for detection by applying waveform A on a disposable gold working electrode with a pH-Ag|AgCl reference electrode in the electrochemical detector. A 10 mM NaOH mobile phase, achieved by proportioning four eluents, at a flow rate of 0.5 mL min^−1^ was used to isocratically separate the four carbohydrates of interest. Mannitol (Sigma-Aldrich, ≥98%), glucose (Tokyo Chemical Industry, >98.0%), sucrose (Fisher Scientific, >99%), and fructose (Sigma-Aldrich, ≥99%) were dissolved in ultrapure water to make carbohydrate standards that were run to create six-point or more calibration curves, with *R*^2^ values ≥0.995, ranging from 0.003 to 5.00 mg L^−1^. Batches included eight to sixteen samples, with spikes (0.31 mg L^−1^) and lab blanks run once for every eight to sixteen samples and field blanks run once for every five samples. Spike recoveries were monitored to ensure they were within ±20% of the actual concentration (Table S1), and additional check standards were run with each batch to ensure standard recovery within ±10%. Limits of detection were determined as three times the standard deviation of seven replicate injections of the lowest concentration standard (Table S1).

#### 2.5.4 Endotoxin analysis

Atmospheric samples (filter quarter) and rain samples were analyzed for endotoxin using the *Limulus* Amebocyte Lysate (LAL) assay. Sample extraction is explained in detail by Sauvé *et al.*,^101^ with extract analysis for endotoxin described previously by Thorne.^102^ Filters were extracted into 3.00 mL of LAL reagent water at room temperature through a process of shaking (30 min), sonication (30 min, 26 °C), shaking (10 min), and finally centrifugation (5 min, 600×*g*, 4 °C). The filter extracts were then analyzed for endotoxin at three dilutions with the LAL assay.^102^ Absorbance at 405 nm (SpectraMax Plus 384; Molecular Devices) was measured and converted to concentrations with a 12-point curve generated for *Escherichia coli* 055:B5 standard endotoxin. Results are reported with units of endotoxin units (EU), which are a measure of endotoxin activity.

## 3 Results and discussion

### 3.1 Campaign overview

**Fig. 1:**
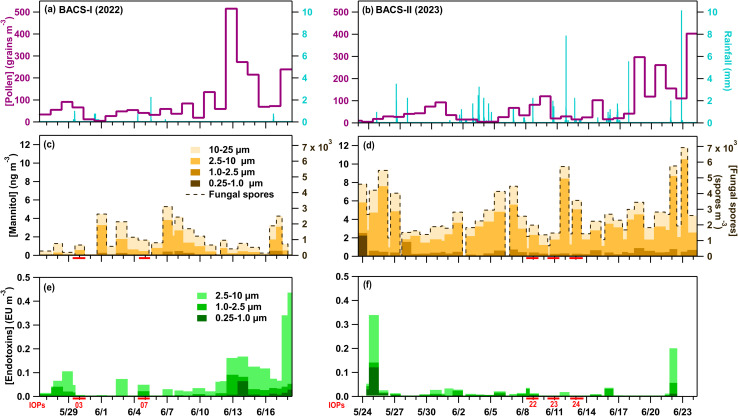
Average daily pollen concentration (dark pink), and rainfall amount (teal) from the National Atmospheric Deposition Program for BACS-I (a) and from the soil plot meteorological station summed over 15-minute intervals for BACS-II (b); mannitol for high flow impactor (HFI) samples across stages from 0.25–25 µm and fungal spores derived from mannitol (1–25 µm) for BACS-I (c) and BACS-II (d); endotoxin for HFI samples across stages from 0.25–10 µm for BACS-I (e) and BACS-II (f). Additional chemical tracer and meteorological time series are included in the SI as Fig. S1 and S2.

The BACS-I and -II field campaigns were characterized by highly contrasting atmospheric environmental conditions and aerosol populations. BACS-I featured “severe drought” conditions (US Drought Monitor), with only 10.1 mm of precipitation over 26 days, while the BACS-II campaign was conducted during one of the wettest summers on record for most of the Colorado plains, with 118.6 mm of precipitation over 33 days (Fig. 1). A total of 23 and over 30 convective cold pools were observed during BACS-I and -II, respectively, with associated convective storm morphologies including multicellular, scattered, and squall line storm systems. Despite higher peak pollen concentrations for BACS-I (516 grains m^−3^) than BACS-II (403 grains m^−3^) (Fig. 1a and b), average log transformed pollen concentrations were not significantly different (*p* = 0.2). Sucrose and fructose had low detection frequencies for BACS-I and -II, with higher concentrations typically reached during warm, dry periods (Fig. S1d, e, S2d and e). Pollen types for both campaigns were dominated by pine (*Pinus*), mountain cedar (*Juniperus*), weed, and grass. Average endotoxin concentrations were significantly greater during BACS-I, based on log transformed data (*p* < 0.01), with greatest concentrations during warm, often dry, periods (Fig. 1e and f).

Mannitol concentrations for particles from 1–25 µm were significantly higher for BACS-II (Fig. 1d) than for BACS-I (Fig. 1c), based on log transformed data (*p* < 0.01). Additionally, for BACS-II, the fraction of mannitol from 1–10 µm was significantly greater for rain/post-rain samples compared to dry samples based on log transformed data (*p* < 0.01). Fungal spores for both campaigns were mainly dominated by spores from *Cladosporium* and *Alternaria*, and by ascospores. For BACS-II alone, likely smut spores had high concentrations, especially during convective events. This observation is supported by reports in May of 2023 of crop diseases caused by smut and rust spores appearing in Colorado, with the growth of these spores attributed to the increased rainfall leading to conditions favorable for the fungal diseases.^103^ Unlike the wetter BACS-II, smut and rust crop diseases were not reported in Colorado during BACS-I. These fungal spore trends align with changes in weather between campaigns, with greater overall fungal spores for the more humid, and wet campaign (BACS-II).

**Fig. 2:**
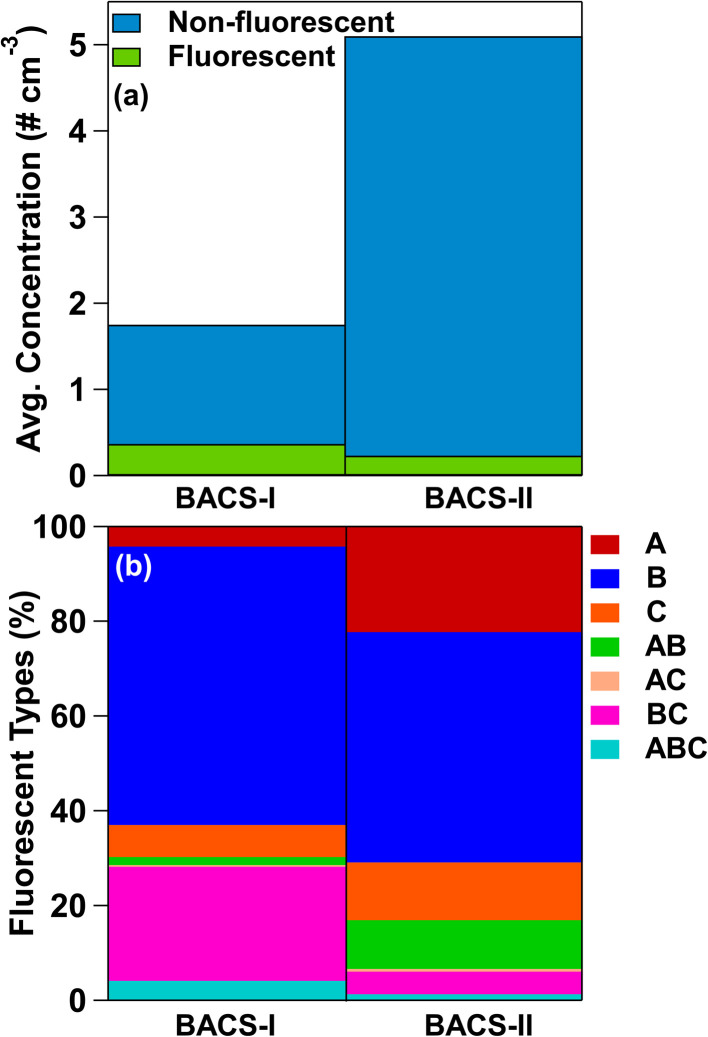
Campaign averages for (a) fluorescent particle and total particle concentrations, and (b) fractions of fluorescent particle types.

Measured fluorescent particles (FPs) by the WIBS-5 reveal unique bioaerosol populations between the campaigns, with higher average non-fluorescent and fluorescent particle number concentrations and FP number fractions at sizes larger than 0.5 µm for BACS-I than for BACS-II (Table 1 and Fig. 2). Larger contributions from supermicron A- and AB-types during BACS-II, previously associated with fungal spores,^76–78^ align with greater mannitol concentrations for BACS-II relative to BACS-I. Fluorescent particle concentrations typically demonstrated a diurnal cycle during BACS-II, with a less clear cycle during BACS-I, with supermicron concentrations peaking overnight into the early morning, coinciding with a decreased atmospheric boundary layer depth (Fig. S3 and S4). Fungal spores likely contribute to this diurnal trend, as concentrations often increase overnight due to environmental conditions favoring active emission and a stable boundary layer that enhances surface concentrations.^104,105^ Cold pools (CPs) and precipitation from convective storms produced a complex variety of responses in bioaerosols across both campaigns, which are explored through case studies from BACS-I and -II.

**Table 1:** Overview of atmospheric conditions, online, and offline measurements for BACS-I and -II, where BDL is below detection limit^a^

	BACS-I (May 23–June 17, 2022)		BACS-II (May 22–June 23, 2023)	
Total precipitation^b^ (mm)	10		119	
Samples with rain	5 of 26		20 of 33	
Cold pools observed^c^	23		33–37	

	**Avg. (*σ*)**	**Range**	**Avg. (*σ*)**	**Range**
Temperature (°C)	17.1 (8.6)	0.4–38.5	16.1 (2.4)	6.9–29.2
RH (%)	50.3 (25.8)	5.4–101	73.7 (16.0)	24–95
Pollen (grains m^−3^)	87 (113)	5–516	72 (91)	5–403
Endotoxin (EU m^−3^)^d^	0.3 (0.3)	BDL – 1.2	0.07 (0.10)	BDL – 0.42
Mannitol (ng m^−3^)^d^	2.6 (1.8)	BDL – 6.0	5.8 (2.6)	2.1–12.2
Glucose (ng m^−3^)^d^	11.7 (12.2)	BDL – 43.9	38.4 (25.9)	10–144
Sucrose (ng m^−3^)	7.7 (12.2)	BDL – 62.4	4.3 (4.2)	BDL – 13.6
Fructose (ng m^−3^)	1.4 (1.4)	BDL – 6.2	1.8 (1.5)	BDL – 6.3

**WIBS-5 particle types**				
Fluorescent (cm^−3^)	0.4 (0.4)		0.2 (0.3)	
Fluorescent (% total)	21.4 (6.9)		6.2 (3.1)	
A (% FP)	4.2 (4.2)		22.3 (12.2)	
B (% FP)	58.8 (13.2)		48.6 (16.1)	
C (% FP)	6.8 (5.4)		12.2 (8.4)	
AB (% FP)	1.8 (3.4)		10.3 (8.5)	
AC (% FP)	0.2 (0.9)		0.6 (1.9)	
BC (% FP)	24.1 (9.5)		4.8 (4.6)	
ABC (% FP)	4.1 (4.6)		1.3 (3.4)	

a Concentrations of chemical tracers are reported for particles <25 µm, <10 µm for endotoxin, including the <0.25 µm after-filter.

b Total precipitation for both campaigns was measured with the N-CON rainwater collector.

c Cold pools characterized during intensive operation period (IOP) days.

d Averages between campaigns are significantly different at the 95% confidence interval for data that was normally distributed after log-transformation; this comparison was not applied to meteorology or fluorescent particle measurements.

A WIBS-4a instrument was also operated during the BACS-I and BACS-II campaigns at the Semi-Arid Grassland Research Center (SGRC) allowing for a spatial comparison of average campaign fluorescent particle concentrations and profiles. The WIBS-4a demonstrated similar trends to WIBS-5 during both campaigns for campaign averages, including higher average FP fractions during BACS-I compared to BACS-II (Table S2). Like WIBS-5, the relative abundance of FPs measured by the WIBS-4a shifted toward greater contributions from A- and AB-types during BACS-II compared to BACS-I (Table S2). The agreement of WIBS-4a and WIBS-5 located ∼3 km apart demonstrates the FP trends observed at SGRC and CPER are consistent on this spatial scale in both campaign years.

### 3.2 Case studies of convective storms

Case studies of convective storms were chosen from intensive operation periods (IOPs) across the two BACS campaigns based on convective events and available measurements. IOPs featuring cold pools with clear thermodynamic and wind signals were prioritized for analysis and were narrowed down based on the availability and quality of sampled data. This included the availability of chemical tracer data and ice nucleating particle (INP) spectra for cases from BACS-I and -II, and continuous flow diffusion chamber (CFDC) data from BACS-II. The case studies discussed here provide examples of cold pools with some of the largest temperature deficits observed during the campaigns, instances of significant fluorescent particle responses, and cases conducive to INP comparison. The subsequent discussion of cold pool impacts on bioaerosols therefore represents, if positively correlated, an upper limit for the IOPs from the BACS campaigns. For each case study, an overview of the atmospheric and convective conditions is presented before a discussion of online measurements (WIBS, CFDC), followed by offline measurements of chemical tracers, intact pollen and fungal spores, and INPs. Due to more observed cold pools and precipitation, case studies from BACS-II are first presented and discussed, followed by BACS-I case studies and comparison.

#### 3.2.1 IOP22 – June 8, 2023

**Fig. 3:**
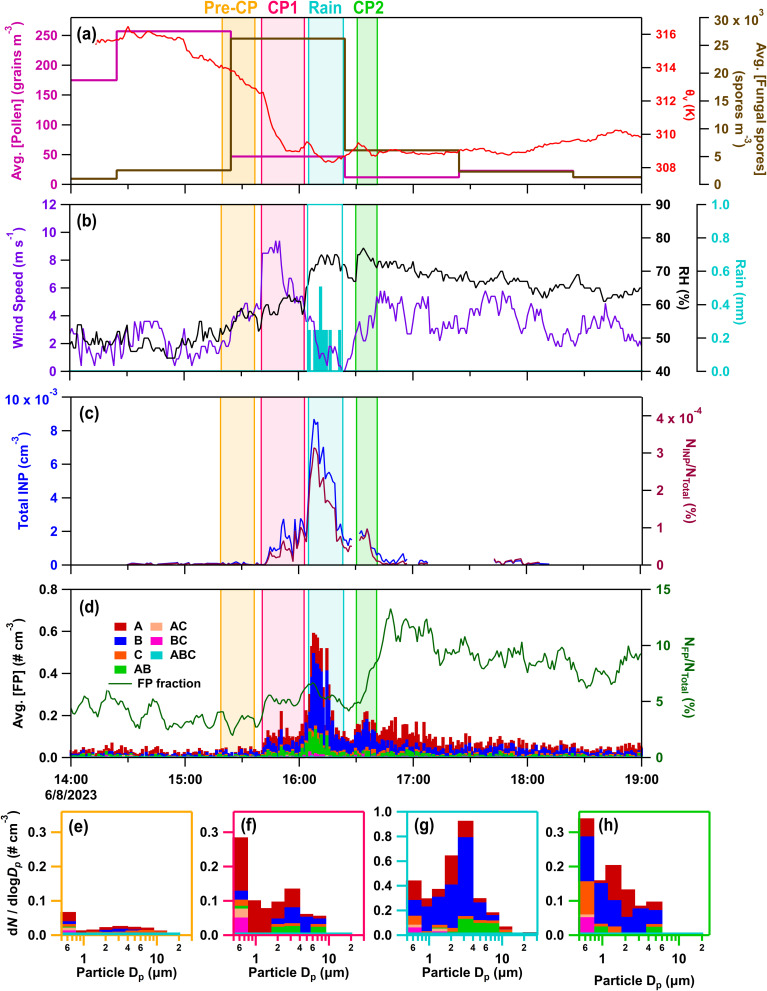
Meteorological and aerosol data from June 8, 2023 (IOP22), with pre-cold pool (CP) (yellow), CP1 (light pink), rain (blue), and CP2 (green) periods outlined. (a) Average pollen concentration (pink), fungal spore concentration (>5 µm) (brown), and virtual potential temperature (red); (b) wind speed maximum for one minute period (purple), relative humidity (RH, black), and rain (blue); (c) INP concentration per standard cm^−3^ (dark blue) and fraction (dark red) active at −20 °C derived from the immersion mode continuous flow diffusion chamber (CFDC) measurements and collocated condensation particle counter (CPC) measurements; (d) stacked average fluorescent particle (FP) concentrations per standard cm^−3^ for seven types (A, B, C, AB, AC, BC, ABC) and the fraction of FPs using the WIBS fluorescent and total measured particles (five-minute moving average) (dark green). Stacked FP type size distributions, with the time averaged in parentheses, during (e) pre-CP period (15:18–15:38), (f) CP1 (15:40–15:56), (g) rain (16:05–16:21), and (h) CP2 (16:30–16:40).

On June 8, 2023 (IOP22), a series of two cold pools, with rainfall in between, occurred and were characterized (Fig. 3a). The first cold pool (CP1) propagated from convection occurring to the west of the CPER site (∼15:40–16:00), altering virtual potential temperature (Δ*θ*_v_), which is the temperature needed for a dry parcel of air to have equal density to a moist parcel while accounting for altitude changes in temperature, relative humidity (ΔRH) and peak horizontal wind speed (Δ*U*) (Δ*θ*_v_ = −3.8 K, ΔRH = −4.2%, Δ*U* = 5 m s^−1^). CP1 increased peak one-minute average fluorescent particle number concentrations (6.5×) and fractions (2×) relative to a ten-minute average of the pre-cold pool values (Fig. 3d). The following 2.76 mm of precipitation (16:05–16:21), with a peak rate of 0.77 mm min^−1^, increased the peak FP concentration by over 16× relative to pre-cold pool levels. The second cold pool (CP2) (Δ*θ*_v_ = −0.2 K; ΔRH = 6.5%; Δ*U* = 5.0 m s^−1^), coming from convection occurring to the north of the site (∼16:30–16:40), kept FP number concentrations enhanced relative to pre-cold pool levels (2.0×), while substantially increasing the fluorescent particle fraction (3×). Offline high flow impactor (HFI) samples were collected on IOP22 for pre-rain (8:37 to 15:00) and rain/post rain (15:00 to 11:34 the following day) periods (Table S3). All CP and rain activity was captured in the rain/post rain offline sample.

Immersion mode INP concentrations from online CFDC measurements at −20 °C greatly increased during all three convective events, with peak concentrations reached during precipitation (>120× relative to pre-cold pool levels) (Fig. 3c). The INP fraction, the concentration of aerosol particles active as INPs divided by a collocated condensation particle counter (CPC) fraction, increased by over 18× relative to the pre-cold pool background during this time as well. This indicates the influence of a new aerosol source rich in INPs, rather than a proportional enhancement of the aerosol population that existed pre-cold pool. INP concentrations and fractions decreased rapidly following CP2, in contrast to the more slowly decaying concentrations of FPs. The slow reduction in FPs led to an increase in their fraction, indicating the release of new sources that were not INPs.

Fungal spores increased markedly during convection, with loading of spores (>5 µm) increasing by 10× relative to pre-cold pool levels during CP1 and the period of rainfall. During the hour of CP1 and precipitation, the most prevalent spore type was identified as smut spores (*Ustilaginomycetes*) (Fig. S5),^94,106^ which are plant pathogens that primarily affect grasses and have shown some ice nucleating ability.^54^ From 15:20–16:20, smut spores with diameters 7 to 15 µm were the predominant bioaerosol type observed by microscopy and their concentrations amounted to 60% of the A- and AB-type FPs measured by the WIBS from 2.5 to 25 µm. Enhanced fungal spore concentrations are further supported by increases in mannitol for the rain/post-rain sample (1–10 µm) (Fig. 1e), and in FPs associated with fungal spores (A- and AB-types from 2–15 µm).^73,76,77^ Mechanisms for fungal spore enhancement could include transport by convective cold pool winds, active ejection with increased relative humidity,^26^ and dispersal through rain induced suspension of spores from surfaces.^15,21,107^ Non-biological fluorescent particles were also not expected to contribute substantially to the WIBS signal, for which the majority was attributed to smut spores. Bacteria and pollen were not expected to contribute significantly to WIBS FPs, based on low concentrations of bacterial endotoxins (Fig. 1f) and pollen tracers (Fig. S2d and e).

**Fig. 4:**
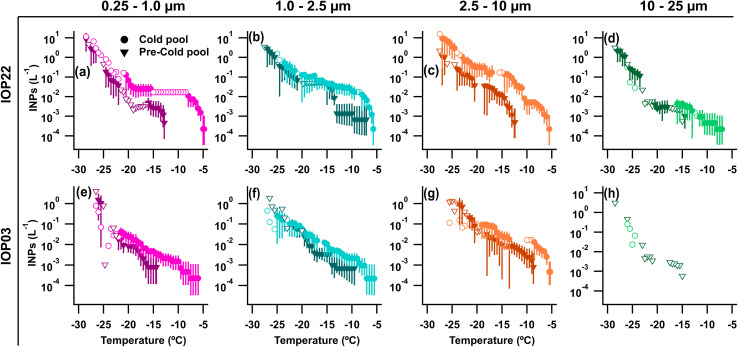
INP analysis for high flow impactor (HFI) samples accumulated for ∼24 hours total between the two samples for each IOP. Samples are split into before the cold pool and precipitation (Pre-CP) events (inverted triangles) and from the sample collected during the cold pool passage and precipitation (CP) (circles) occurrence through the sample change the following morning. Exact accumulation times are available in the text as well as Table S3. INPs across four stages (0.25–1.0 µm, 1.0–2.5 µm, 2.5–10 µm, 10–25 µm) are included for (a–d) IOP22, and (e–h) IOP03. Error bars represent the 95% confidence interval. Hollow markers represent non-significant data points at the 95% confidence level.

Warm-temperature (>−15 °C) heat-labile INPs sampled during the convective events of IOP22 and the following night increased across all measured size ranges in the rain/post-rain HFI sample, particularly for particles across three stages for sizes from 0.25–10 µm (Fig. 4a–d and S6a–d). Following heat treatment of the rain/post-rain sample, over 90% of INPs for temperatures >−15 °C were deactivated in 0.25–10 µm particles (Fig. S6a–c), supporting bacterial and fungal contributions to enhanced INPs during and following rainfall. This is in line with conclusions from earlier studies that attribute the increase of warm-temperature INPs during rainfall to fungal spores and/or bacteria.^15,25,61,108,109^ Huffman *et al.*^15^ in particular, observed an increase in INPs and FPs in the 2–6 µm size range during rainfall, which they attributed to fungal spores and bacteria. Here, increases of INPs from 1–10 µm align with enhanced mannitol concentrations and A- and AB-type FPs, suggesting significant fungal contributions to these INPs. Bacterial contributions are expected to be minor compared to fungal spores as endotoxin in the rain/post-rain sample is greatly reduced in particles from 1–2.5 µm, with only small increases in the 0.25–1, 2.5–10, and 10–25 µm stages (Fig. 1f). In the pre-rain sample, a large population of heat-labile INPs in the 1–2.5 µm size range have a sharp activation at −14 °C (Fig. 4b and S6b Pre-CP). This sharp activation indicates a substantial population of uniform INPs consistent with characteristics of a single biological ice nucleating entity. Note that, the endotoxin distribution shows high levels in the pre-rain sample and lower levels in the rain/post-rain sample. Higher time resolution measurements by Mignani *et al.* during BACS-I and -II at the SGRC before, during, and after rain demonstrated that active rainfall is responsible for enhanced INP concentrations.^25^

#### 3.2.2 IOP23 – June 10, 2023

**Fig. 5:**
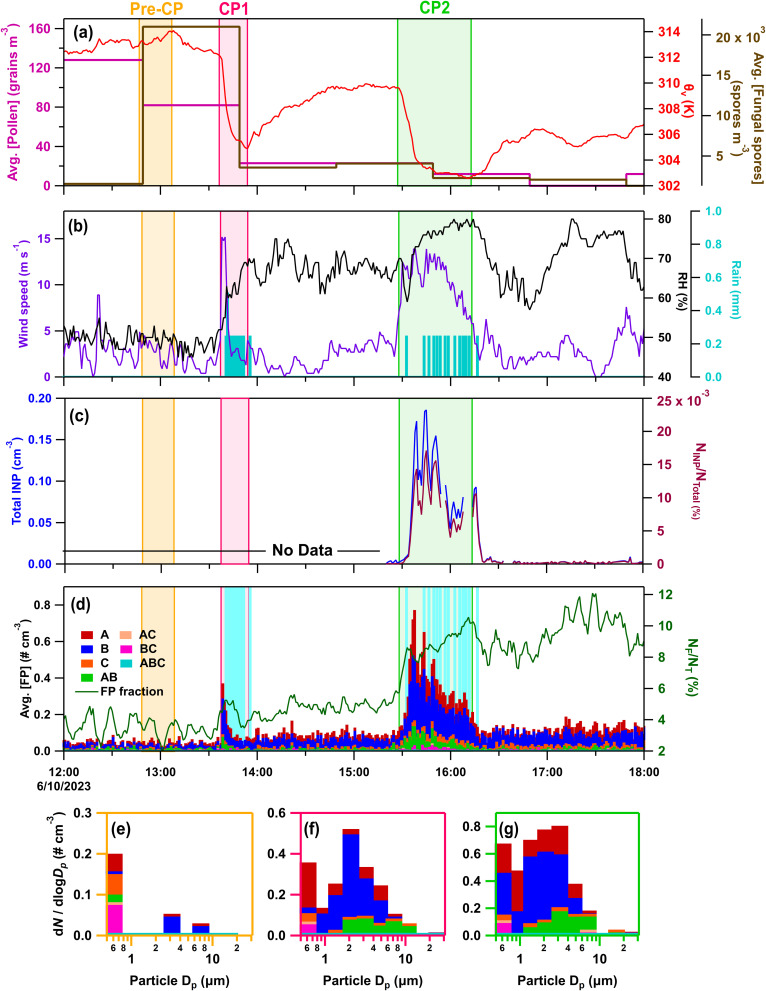
Online data from IOP23 on June 10, 2023. Panels (a–d) as in Fig. 3a–d. Stacked FP type size distributions, with the time averaged in parentheses, during (e) pre-cold pool (CP) period (12:47–13:07) (f) CP1 (13:36–13:50), (g) CP2 (15:28–15:48).

On IOP23 (June 10, 2023) two convective events passed through the site (Fig. 5). CP1 (∼13:36–13:55) was interspersed with 3.51 mm of precipitation with a peak rate of 0.47 mm min^−1^. Coming from the northwest, with larger magnitude thermodynamic and wind speed perturbations compared to other case studies (Δ*θ*_v_ = −7.6 K; ΔRH = 26.8%; Δ*U* = 14.2 m s^−1^) (Fig. 5a and b), CP1 greatly increased FP peak concentrations (7.9×) and fractions (2×) relative to pre-cold pool levels (Fig. 5d). CP2 (∼15:28–16:11), produced by a squall line system, passed over the site in association with 3.75 mm of precipitation at a peak rate of 0.21 mm min^−1^. CP2 came from the west-southwest (Δ*θ*_v_ = −6.7 K; ΔRH = 13.7%; Δ*U* = 10.1 m s^−1^), again increasing both FP peak concentrations (9.7×) and fractions (2×). Offline HFI samples were collected for the pre-rain period from 08:40 to 13:37 and the rain/post-rain period from 13:37 to 12:16 the next day, which captured all CP and rain activity. These were not analyzed using the Ice Spectrometer (IS). CFDC online INP data, available only for CP2, indicate significantly enhanced INP concentrations and fractions active at −20 °C during CP2 relative to pre- and post-cold pools (>40×). The peak INP concentration of ∼0.2 standard cm^−3^ at −20 °C was the largest among all the considered case studies, and exceptionally high compared to longer-averaged INP measurement in terrestrial sites.^110^ Following the cold pool and rain window, concentrations and fractions of INPs decayed more rapidly than FPs (Fig. 5c and d), similarly to IOP22 (Fig. 3c and d), supporting the interpretation that post-rainfall bioaerosol composition shifts toward aerosol species that are not IN active at warm temperatures, consistent with findings by Mignani *et al.*^25^

Hourly fungal spore counts by microscopy indicated increases in fungal spores (>5 µm), primarily smut spores (Fig. S5), concurrent with cold pools and precipitation. Notably, the loading of smut spores increased much more significantly (13×) relative to pre-rain background during CP1 compared to CP2 (1.2×), indicating likely differences in fungal spore sources between CP1 from the northwest and CP2 from the west-southwest. Increases in fungal spores during the cold pools and precipitation were also indicated by increases in mannitol in particles from 1–10 µm (24-h sample) (Fig. 1e), and by increases in A-, and AB-type FPs ranging in diameters from 1–20 µm (Fig. 5e and f). A lack of endotoxin and pollen tracers from the 24-h sample, collected during the convective storms, further supports fungal spores as the dominant bioaerosol type during these two cold pool and precipitation events.

#### 3.2.3 IOP24 – June 12, 2023

**Fig. 6:**
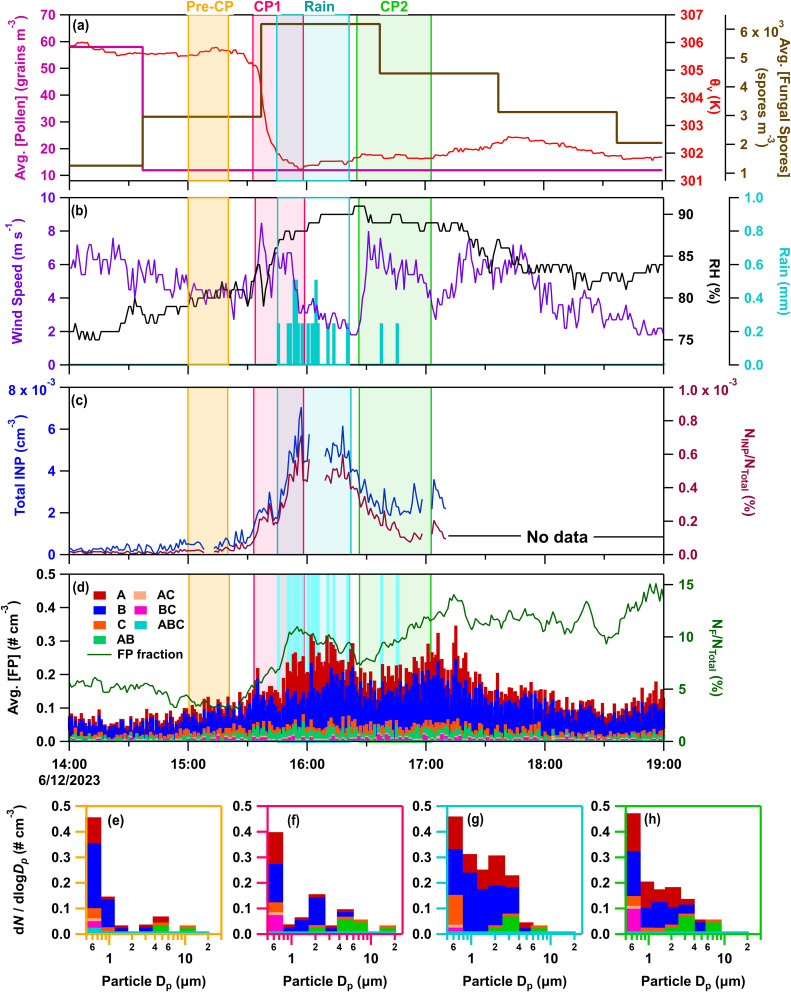
Online data from IOP24 on June 12, 2023. Panels (a–d) as in Fig. 3a–d. Stacked FP type size distributions, with the time averaged in parentheses, during (e) pre-cold pool (CP) period (15:00–15:20), (f) CP1 (15:32–15:47), (g) rain (15:50–16:05), and (h) CP2 (16:28–16:43).

On IOP24 (June 12, 2023), two cold pools passed through the CPER site with precipitation between their passage. CP1 (∼15:32–16:00) advanced from the southeast (Δ*θ*_v_ = −4.0 K; ΔRH = 7%; Δ*U* = 4.4 m s^−1^) (Fig. 6a and b), moderately increasing FP number concentrations (2.1×) and fractions (2×) before rainfall relative to pre-cold pool levels (Fig. 6c). Intermittent rain followed, totalling 4.5 mm of rain from 15:45 to 16:20 (at a peak rate of 0.61 mm min^−1^), further increasing FP number concentrations (2.7×) and fractions (2.6×) compared to the pre-cold pool environment. CP2 (∼16:26–17:05) was a thermodynamically weak cold pool that passed through from the northwest (Δ*θ*_v_ = −0.05 K; ΔRH = −1.5%; Δ*U* = 5.5 m s^−1^) with little temperature change relative to conditions before its passage, but with a signal in wind direction and speed. It maintained enhanced FP concentrations and increased the FP fraction (1.7×). Offline HFI samples were collected for a pre-rain period from 08:25 to 15:35, and a rain/post-rain period from 15:35 to 12:11 the next day, which captured all CP and rain activity. Measurements of INPs from the CFDC showed enhanced INP concentrations and fractions during both cold pools, with rainfall increasing concentrations by >4× relative to pre-CP levels. During CP2, INP number fractions decline while FP number fractions increase, indicating that the bioaerosols enhanced in CP2 are less INP active. This trend contrasts CP1 in IOP24, and IOPs 22 and 23, where similar trends in INP and FP number concentrations and fractions suggest the same or a similar source. The divergence between INPs and FPs during CP2 on IOP24 captures the variability of CP impacts on bioaerosols and INPs that is not observable in the offline measurements.

Increasing hourly fungal spore counts during CPs and rain (Fig. 6a), along with increased mannitol (Fig. 1e) and A- and AB-types for particles 1–10 µm (Fig. 6f–h) support fungal spores dominating the enhanced bioaerosol signal. A lack of detectable endotoxin, and fructose and sucrose in HFI samples indicate little influence from intact pollen, pollen fragments, and Gram-negative bacteria (Fig. S2d, e and 1f). Although INP samples were not heat treated for IOP24, INPs measured across all HFI sample sizes for the pre-rain and rain/post-rain samples exhibit warm-temperature INPs with spectral shapes strongly indicative of major heat-labile biological components (Fig. S6e–h). The offline rain/post-rain sample demonstrates enhancement of INPs from 0.25–2.5 µm by nearly one order of magnitude compared to pre-rain levels for these likely biological components, with the majority of INPs found in the 1–2.5 µm range.

#### 3.2.4 IOP03 – May 29, 2022

**Fig. 7:**
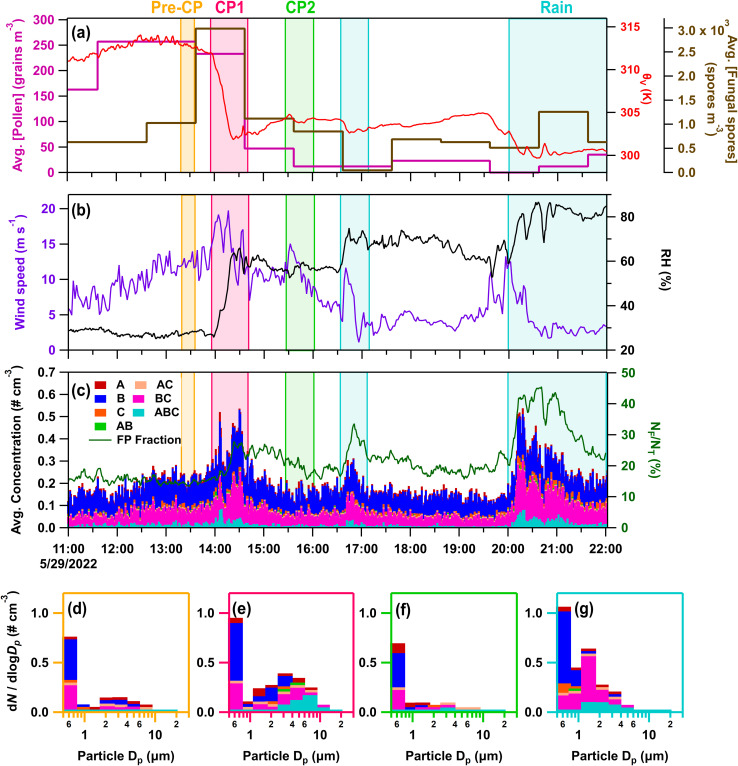
Online data from IOP03 on May 29, 2022. Panels (a–c) as in Fig. 3a, b and d. Stacked FP type size distributions, with the time averaged in parentheses, during (d) pre-cold pool (CP) period (13:19–13:35), (e) CP1 (13:58–14:12), (f) CP2 (15:30–15:44), and (g) rain (20:10–20:23).

The next two case studies focus on convective storms observed during BACS-I, which was much warmer and drier overall compared to the BACS-II campaign. On IOP03 (May 29, 2022), a series of two cold pools passed through the CPER site over a two-hour period. CP1 (∼13:58–14:50) was produced by a squall line system to the west of the site, resulting in the largest temperature perturbation observed during BACS-I (Δ*θ*_v_ = −10.2 K; ΔRH = 39.1%; Δ*U* = 6.4 m s^−1^) (Fig. 7a and b). Fluorescent particle number concentrations and fractions more than doubled during the first cold pool's passage relative to pre-cold pool concentrations (Fig. 7c). CP2 (∼15:26–16:02) passed from the west, with much smaller temperature and moisture perturbations (Δ*θ*_v_ = −0.2 K; ΔRH = 3.8%; Δ*U* = 3.6 m s^−1^) (Fig. 7a and b), and did not substantially alter local bioaerosols. Rainfall occurred from ∼16:30 to 17:00 and ∼20:00–22:00 (2 mm total), increasing both FP number concentrations and fractions relative to pre-rain levels, particularly for the second event which increased them by 4.0× and 2.5×, respectively. HFI samples were collected for the pre-rain period from 08:23 to 14:07 and for the rain/post-rain period from 14:07 to 10:57 the next day, capturing both CPs and the rain events, aside from the first few minutes of CP1.

During IOP03, fungal spores were enhanced by the first cold pool and rain events based on increases in the hourly fungal spore counts (>5 µm), increasing by 3× during CP1 and by 2.5× during the later precipitation, which were dominated by *Cladosporium* spores and ascospores (Fig. 7a). Enhanced fungal spores during convection are also supported by increases in rain/post-rain mannitol for particles 2.5–10 µm (Fig. 1b) and increases in ABC-type FPs from 1–10 µm. Meanwhile, Gram-negative bacteria and pollen were ruled out as major sources of FPs due to a lack of detectable pollen tracers (Fig. S2d and e), and a decrease in endotoxin from the pre-rain sample in the size range from 1–10 µm (Fig. 1c). Despite increased FP and fungal spore concentrations, warm-temperature INP concentrations were relatively low across size and temperature ranges for both total and heat-labile INPs, with a minor increase in the CP and rain sample that is most evident >−15 °C (Fig. 4e–h and S6i–l). This further illustrates that total bioaerosol concentrations are not always predictors or even strongly correlated with INP concentrations, due to the likely small subset of biological species that produce efficient IN materials. This is discussed in more detail in Section 3.3 using correlation analysis.

#### 3.2.5 IOP07 – June 4, 2022

**Fig. 8:**
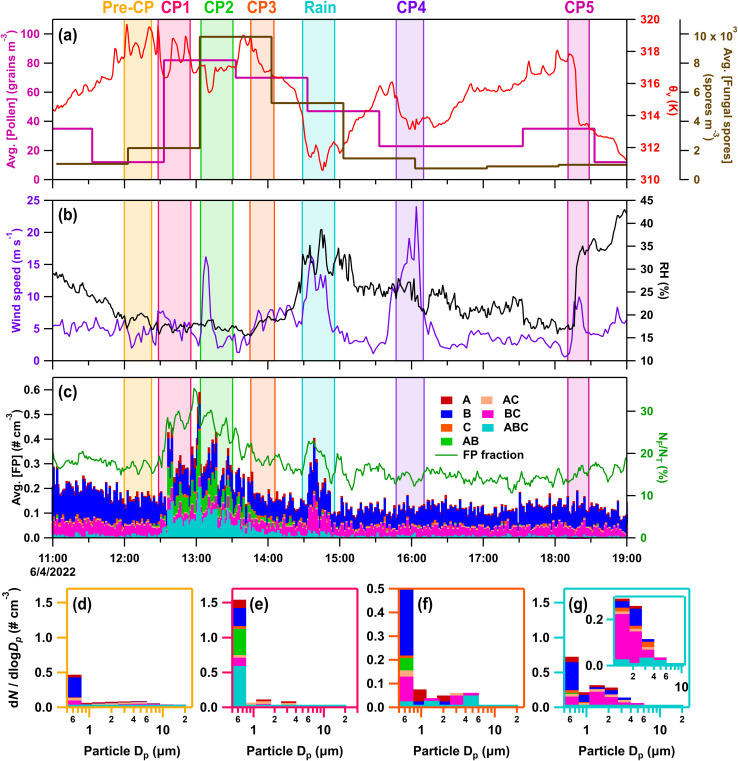
Online data from IOP07 on June 4, 2022. Panels (a–c) as in Fig. 3a, b and d. Stacked FP type size distributions, with the time averaged in parentheses, during (d) pre-cold (CP) pool period (12:00–12:23), (e) CP1 (12:36–12:51), (f) CP3 (13:46–14:06), and (g) rain (14:33–14:48).

On IOP07 (June 4, 2022), five cold pools passed through the CPER site along with a precipitation event. CP1 (Δ*θ*_v_ = −2.5 K; ΔRH = −2.1%; Δ*U* = 4.0 m s^−1^) and CP2 (Δ*θ*_v_ = −1.5 K; ΔRH = 1.0%; Δ*U* = 12.5 m s^−1^) (Fig. 8a,b) passed through the site from ∼12:30–13:30, with winds from the west-southwest and the east, respectively. The passage of CP1 increased fluorescent particle number concentrations (3.2×) and fractions (2.1×) relative to pre-cold pool levels, primarily A-, AB-, and ABC-types from 0.5–0.75 µm (Fig. 8e). These particles remained enhanced during CP2, but decreased during CP3 from the northeast (Δ*θ*_e_ = −2.0 K; ΔRH = 1.7%; Δ*U* = 5.3 m s^−1^) (Fig. 8f). Rainfall followed (∼14:30–14:50) with 1.6 mm of rain increasing FP number concentrations by 3.5× pre-rain levels, primarily enhancing B-, BC-, and ABC-type particles from 1–6 µm (Fig. 8g). CP4 and CP5 had essentially no impact on fluorescent particle number concentrations, despite cold pool-induced thermodynamic perturbations (Table S4). HFI samples were collected from 08:22 to 08:18 the following day.

Increases in fungal spore counts during CPs 2–3 and rainfall suggest fungal spores were impacted by the storms, as does mannitol for particles from 2.5–25 µm (Fig. 1b), but these increases were not reflected in FPs during CPs 2–3. Bacterial endotoxin from 0.25–10 µm suggest some contributions of bacteria to enhanced bioaerosols, while a lack of detected pollen tracers suggest little pollen influence. The first two cold pools greatly increased number concentrations of AB- and ABC-types from 0.5–0.75 µm to >20× background levels, with similar profiles also observed on additional days during BACS-I (May 27, 2022 – IOP02; June 7, 2022 – IOP09; June 8, 2022). The fluorescent profile suggests the signal to be uniquely biological, but it is not fully explained by chemical tracers or previous lab studies.^72,76,77,111^

Abundant heat-labile, warm-temperature (>−15 °C) INPs were measured for particles 0.25–10 µm (Fig. S6m–p), with greatest concentrations for particles 2.5–10 µm (Fig. S6o). These heat-labile INPs accounted for >99% of INPs active at temperatures warmer than −20 °C in the 0.25–2.5 µm range, and >90% in the 2.5–10 µm range. Mannitol concentrations peaked in particles 2.5–10 µm for the collected sample (24 h), as did ABC-types during rain, suggesting potential contributions from fungal spores to these INPs.

### 3.3 Evaluation of FPs as markers for biological INPs

Correlation analysis was applied to examine the relationship between FPs and INPs, using ice spectrometer measurements applied to four samples from BACS-I and five samples from BACS-II, including IOPs 3, 7, 22, and 24. The samples analyzed for offline INPs were all from IOPs, and contained both pre-rain and rain/post-rain samples. FP concentrations were averaged over the sample time of the offline INP measurements (6–24 h). Comparisons between FPs with offline INPs reveal significant differences in correlation between particle size, with significant positive and negative correlations for particles from 1–2.5 µm, and weak (insignificant) correlations for particles from 2.5–10 µm (Table S5). Significant correlations of FP types with offline INP concentrations are found at −20 °C in the 1–2.5 µm size fraction, with positive correlations for A-, B-, and AB-type particles (*r* = 0.79, 0.70, and 0.84 respectively at *p* < 0.05), negative correlations for BC-, and ABC-type particles (*r* = −0.76, and −0.82 respectively at *p* < 0.05), and no correlation with the total FP concentrations (*r* = −0.02 at *p* > 0.05) (Table S5). Correlations between FP and offline INP concentrations are weaker and less significant with measurements in the 2.5–10 µm size range, with significant correlations only with B-type particles (*r* = 0.74, *p* < 0.05), demonstrating the sensitivity of this analysis to particle size. Differences in the sampling rates between online INP measurements (minute-scale) and offline samples (hourly to daily) likely also contribute to weaker correlations, with stronger correlations expected for measurements with similar sampling rates. Correlations between FP concentrations with offline INP concentrations reveal the complexity of using FPs as a marker for biological INPs, with marked differences between particle sizes, INP activation temperature, and FP-type.

Online CFDC measurements (<2.5 µm, BACS-II) reveal a different correlation pattern to offline INPs, biased by shorter-term sampling (minute scale) surrounding rainfall and cold pool events (Table S6). For online INP measurements, significant correlations were found for IOPs 22, 23, and 24 for periods during cold pools and rain, based on a threshold of the averaged background plus ten times the standard deviation. For IOP22, the strongest correlation is observed between INP and total particle concentrations measured by the WIBS (*r* = 0.87, *p* < 0.01), followed by correlations with FP and B-type particle concentrations (both *r* = 0.82 at *p* < 0.01) (Table S6). IOP23 shows similar correlation with total FPs (*r* = 0.82 at *p* < 0.01), but lower correlation with total particles and B-type particles (*r* = 0.79 and 0.76 respectively at *p* < 0.01) (Table S6). Unlike IOPs 22 and 23, correlations for IOP24 were strongest between A-type particles with online INP concentrations (*r* = 0.67, *p* < 0.01) (Table S6). Significant positive correlations between online INPs with all FP types for IOPs 22 and 23 contrast with the results of the offline INPs from 2.5–10 µm, where correlations were weak or negative. This analysis demonstrates that capturing changes in INPs through FPs as bioaerosol proxies is a complex task, with likely influences from the sampling time and particulate size. The significant differences between correlations likely also stem from IN active bioaerosol species comprising only a minor fraction of total bioaerosol, with FPs or FP-types not always capturing this IN active bioaerosol fraction. These findings highlight compelling targets for future research, including identifying fungal species associated with increased INP concentrations, testing the IN ability of different smut spore species, the association of these fungi with soils or plant surfaces, and the details of spore release.

### 3.4 Comparison of precipitation impacts across BACS-I and -II

With five days of collected rainfall samples during BACS-I and twenty-one days during BACS-II, precipitation during both campaigns generally enhanced supermicron fluorescent particle number concentrations and fractions relative to pre-rain conditions. During BACS-I, rain increased supermicron FP number concentrations relative to pre-convective conditions by an avg. 4.8× (*σ* = 3.0) and FP fractions by an avg. 2.4× (*σ* = 1.0), while during BACS-II rain increased supermicron FP number concentrations by an avg. 4.7× (*σ* = 4.5) and FP fractions by an avg. 1.6× (*σ* = 0.5) respectively. For both campaigns, fungal spore loading and mannitol typically increased during precipitation events, as did supermicron FPs, indicating fungal spores were the primary bioaerosol type impacted by rainfall. The FP-types associated with fungal spores differed between years, with ABC-types from 1–6 µm dominating responses during BACS-I, and A- and AB-types from 1–20 µm dominating responses during BACS-II. Differences in FP-types primarily observed alongside mannitol during BACS-I and -II are likely due to differences in the sampling site environment, highlighted by significant concentrations of smut spores during BACS-II, with little to no detection during BACS-I. Mannitol distributions shifted to smaller sizes for samples during precipitation, with significant differences between the BACS-II log transformed mean 1–10 µm mannitol concentrations in pre-rain *vs.* rain/post-rain samples (*p* < 0.01, *n* = 11). This result agrees with Rathnayake *et al.*,^20^ which demonstrated increases in the fine fraction of mannitol during rain, attributed to differences in fungal spore emission mechanisms and coarse particle scavenging.^20,26,107^ Pollen and Gram-negative bacteria concentrations were generally reduced for both campaigns in samples collected during precipitation based on intact pollen counts, pollen tracers, and endotoxin measurements. A general lack of pollen tracers from 0.25–2.5 µm and fungal spore tracers from 0.25–1.0 µm rule out significant contributions from their fragments that could have formed from rain or humidity induced rupture.^16,31,112–115^

Additionally, INP concentrations increased during periods with rain based on both online, single-temperature CFDC measurements and offline measurements across a full temperature range. Most case studies had relatively uniform distributions of IN material across sizes, which may indicate the internal mixing of fragmented biological materials containing IN active sites with other aerosols. The INP concentrations and fractions measured by the CFDC at −20 °C, capturing <2.5 µm INP, were greatly enhanced during precipitation compared to pre-rain levels. INP concentrations for IOPs 22, 23, and 24 increased by >5×, >14×, and >4× respectively, indicating strong biological INP response even at −20 °C. Offline measurements of very warm-temperature (−10 °C) INP concentrations (0.25–25 µm), were at least an order of magnitude greater for samples during rain and cold pools compared to dry samples, with greatest increases in the 0.25–10 µm size range. Particularly large enhancements in the 2.5–10 µm size range in IS measurements indicate the dramatic changes to INP concentrations in CFDC measurements (<2.5 µm aerosol) during rainfall understate the changes in total and peak INP concentrations at −20 °C. At temperatures above −20 °C, the changes in INP concentrations are even more pronounced. This is consistent with Mignani *et al.*^25^ who examined INP concentrations before, during, and after rainfall at the CPER site. That study reported significant correlations between INP concentrations and rainfall kinetic energy, suggesting that INPs are emitted by raindrop impact on surfaces, likely originating from plant surfaces or soil. Here, similar precipitation-related enhancements were observed for fungal spores based on Burkard slides, mannitol concentrations, and FP-types like supermicron A-, AB-, and ABC-types. For heat-labile INPs across BACS-I and -II cases, a strong correlation was found with mannitol for particles in the 2.5–10 µm size range (*r* = 0.91, *n* = 8, *p* < 0.01), supporting fungal contributions to local biological INPs (Table S5), consistent with Huffman *et al.*^15^ Unlike mannitol, bacterial endotoxin did not seem to have a clear response to rainfall, which would suggest a minor contribution of bacteria to rain-induced FPs and INPs, though further work is merited.

### 3.5 Comparison of cold pool impacts across BACS-I and -II

Cold pools produced more complex changes in bioaerosols compared to precipitation, with impacts ranging from fluorescent particle concentrations increased 19× above pre-cold pool levels, to 60% decreases in fluorescent particle concentrations from pre-cold pool levels. Cold pools increasing FPs approached the site from all directions (Table S4). Cold pools increased supermicron FP number concentrations for 18 of 33 cases considered during BACS-I and 20 of 27 cases considered during BACS-II. Submicron FPs were impacted more strongly by cold pools during BACS-I and dominated the FP responses in selected cases (*e.g.* IOP07). In the case of supermicron fluorescent particles, fungal spores demonstrated greater responses to cold pools than pollen and bacteria, based on FP-types and sizes. During selected cold pool events, enhanced fungal spore concentrations were observed. In BACS-II, these were dominated by likely smut spores (*Ustilaginomycetes*) (Fig. S5), with average diameters ranging from 7–15 µm, and in BACS-I by *Cladosporium*, *Alternaria*, and ascospores. Except for actively wet discharged ascospores, these spores favor dry and windy conditions for release,^116,117^ but can also be suspended through water droplet splashing.^21^ Increased smut spore concentrations during convective storms for the discussed cases could be due to the higher wind speeds present in cold pools, coupled with initial dispersal by rain splashing, as documented in previous research.^107^ Additionally, some species of *Ustilaginomycetes* have been found to act as INPs,^54^ with *Ustilago nigra* spores nucleating ice at temperatures as warm as −10 °C, indicating the potential for the detected spores to influence local INPs.

**Fig. 9:**
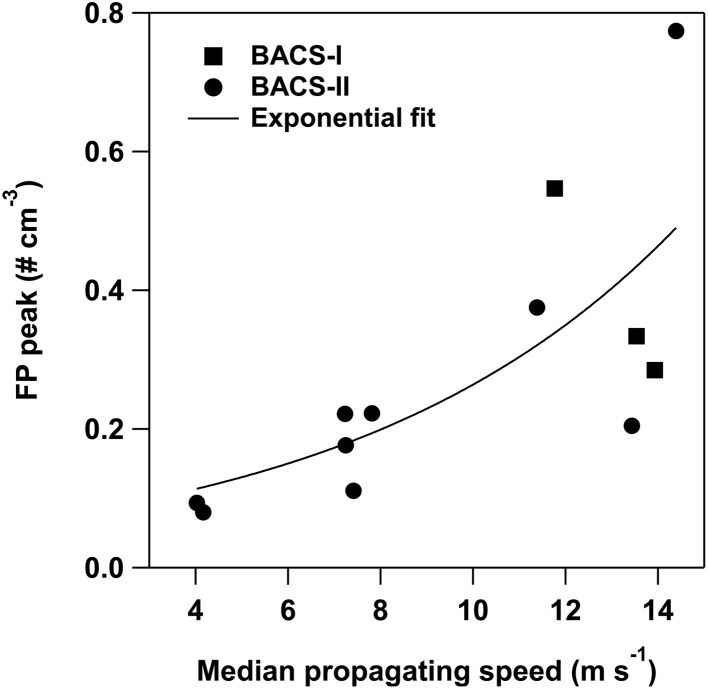
Correlation of peak fluorescent particle concentrations with the median propagating speed of the cold pool determined from radar. Spearman's rank correlation coefficient (*r*_s_) is 0.79 (*p* = 0.03) for combined BACS-I and -II data (*n* = 12), and *r*_s_ = 0.78 (*p* = 0.06) for only BACS-II data (*n* = 9). Data points from BACS-I and -II were fit with the exponential function of *f*(*x*) = 0.06 × exp (0.14 × *x*).

For BACS-II, increases in fluorescent particle concentrations (ΔFP) were typically greatest for cold pools with the largest magnitude thermodynamic and wind perturbations. This is indicated by correlations between cold pool characteristics and changes to FPs across all cases for BACS-II (*n* = 27), which reveal moderate significant correlations between changes in FP concentrations with perturbations in wind speed, temperature, virtual potential temperature, and relative humidity (Table 2). The statistically significant correlation between changes in temperature and FPs during BACS-II demonstrates that CPs with increasing thermodynamic impacts were associated with larger increases in FPs. This association is further supported by a significant correlation between radar-based cold pool propagation speeds,^118^ a robust measure of cold pool strength, and peak FP concentrations, for selected cases across BACS-I and -II with a sufficient CP radar signals to determine CP propagation speeds (Fig. 9). Conversely, during BACS-I, there were no statistically significant correlations between changes in FP concentrations and meteorological perturbations during the cold pool passage (*n* = 33) (Table 2). The lack of significant correlation between cold pool properties and fluorescent particles during BACS-I compared to BACS-II is likely due to the different weather conditions between the campaigns and lower fungal spore concentrations during BACS-I. Limited CFDC data prevented extensive comparison of INPs with cold pool thermodynamic properties, but available data show increased INP concentrations in CPs for all case studies.

**Table 2:** Spearman's rank correlation coefficients (*r*_s_) between fluorescent particles (FPs) and meteorological perturbations of cold pools for BACS-I and -II

	BACS-I (*n* = 33)	BACS-II (*n* = 27)
	ΔFP^a^	ΔFP
Δ Wind speed	0.03	**0.48**
Δ Temperature	−0.13	**−0.47**
Δ Relative humidity	0.17	**0.46**
Δ Equivalent potential temperature	0.10	**−0.41**
Δ Virtual potential temperature	−0.04	**−0.47**

a Delta represents a difference between a pre-cold pool 10-min average and the peak value during its passage. Bolded coefficients have *p* < 0.05.

### 3.6 Comparison of precipitation and cold pool impacts to literature

During the BACS campaign at the CPER site in Colorado, rainfall typically increased bioaerosol and INP concentrations, supporting a growing body of research that demonstrate the increase of bioaerosols by rain.^15,19,20^ Unlike similar studies at a site in Iowa that was near woodlands, grassland, agricultural fields, and suburban areas, where rainfall events were associated with large contributions from pollen fragments,^16,65^ our findings indicate a lack of appreciable pollen rupturing during rain at CPER. Instead, fungal spores appear to be important, as rain primarily enhanced the fungal spore tracer and particle types from 1–10 µm compared to other bioaerosol types. Warm-temperature, heat-labile INPs increased in samples collected during convective storms, with significant correlation of INP concentrations at −15 °C and mannitol concentrations in the 2.5–10 µm size range, indicating fungal spore contributions to INPs. These results are consistent with a study by Conen *et al.*^61^ that demonstrated statistically significant correlations between mannitol and warm-temperature INPs (−8 °C) during rain.

While cold pool impacts varied during BACS, cold pools with larger temperature deficits generally led to greater increases in bioaerosol concentrations. Previous research has primarily been focused on cold pool impacts on dust, with evidence of cold pools lofting dust^119–122^ through the gust front, leading to somewhat limited ingestion of dust by the parent storm.^40,123^ Convective cold pools have been shown to increase INP concentrations, with concentrations in the outflows ranging from 10–100× above pre-storm values.^62^ Our results demonstrate that the cold pool gust front can loft bioaerosols either prior to or in the absence of precipitation, primarily fungal spores with diameters 1–10 µm, with the amount lofted related to the pre-existing bioaerosol environment and cold pool characteristics. Between the two BACS campaigns, fungal spore composition changed dramatically, with an increased presence of smut spores during the wetter BACS-II compared to the drier BACS-I. Unlike fungal spore composition, pollen and Gram-negative bacteria responses remained more consistent between campaigns as assessed from chemical tracers, FP-types, and microscopy in the case of pollen. This study also demonstrates the ability of cold pools to enhance INPs based on CFDC measurements, indicating the potential for ingestion of lofted INPs by the storm, where they could influence precipitation processes. Increases in warm-temperature INPs entrained by the storm could lead to increases in precipitation for midlevel convective clouds,^124^ feeding into a bio-precipitation feedback cycle.^125^ However, INP impacts on precipitation and cloud microphysics are subject to a high degree of uncertainty, and further study is needed to evaluate the impact of biological INPs on precipitation in convective storms.

## 4 Conclusions

Across two campaigns, the characterization of bioaerosols and INPs provides new insights into the impacts of convective storms, particularly cold pools and surface precipitation, on bioaerosols in a grassland environment. Our results demonstrate that convective storms can greatly enhance local bioaerosol concentrations, primarily fungal spores, for the environment studied, through cold pool and surface precipitation events. With their size typically <10 µm, fungal spores may be respired and impart high potential for elevated human exposures to fungal spores during rain and cold pools that accompany convective storms. Convective storms also enhanced warm-temperature, biological INPs during precipitation and cold pools, increasing the number of particles which could impact cloud microphysical properties. To better understand the potential impacts of biological INPs on storms, future studies should focus on improving parameterization of fungal spore transport and emission during convective storms to allow for more accurate modeling of their impacts on convective storms and associated precipitation. Improved understanding of both cold pool and precipitation impacts on bioaerosols in different environments will enable better predictions of potential health hazards, as well as numerical modeling of the influence of bioaerosols as INPs on convective storm dynamics and microphysics.

## Author contributions

Teresa K. Feldman: investigation, formal analysis, visualization, writing – original draft; writing – review & editing; Chamari B. A. Mampage: investigation, writing – review & editing; Nicholas M. Falk: investigation, formal analysis, writing – review and editing; Janeshta C. Fernando: investigation, writing – review & editing; Brian Heffernan: investigation, writing – review & editing; Thomas C. J. Hill: investigation, writing – review & editing; Drew Juergensen: investigation, writing – review & editing; Claudia Mignani: investigation, funding acquisition, writing – review & editing; Marina Nieto-Caballero: investigation, writing – review and editing; Leah D. Grant: conceptualization, funding acquisition, project administration, supervision, writing – review & editing; Susan C. van den Heever: conceptualization, funding acquisition, project administration, supervision, writing – review & editing; Paul J. DeMott: conceptualization, funding acquisition, project administration, supervision, writing – review & editing; Sonia M. Kreidenweis: conceptualization, funding acquisition, project administration, supervision, writing – review & editing; Russell J. Perkins: conceptualization, funding acquisition, project administration, supervision, writing – original draft, writing – review & editing; Elizabeth A. Stone: conceptualization, formal analysis, funding acquisition, project administration, supervision, writing – original draft; writing – review & editing.

## Conflicts of interest

There are no conflicts to declare.

## Supplementary Material

EA-006-D5EA00129C-s001

## Data Availability

Data for this article are available at the National Center for Atmospheric Research Earth Observatory Laboratory Field Data Archive (2025) through https://www.eol.ucar.edu/field_projects/bacs. Data includes pollen counts, chemical tracers, WIBS-5, CFDC, meteorological data for BACS-II, and IS INP measurements. Met data from NEON is available through the NEON data portal (https://data.neonscience.org/data-products/explore). Supplementary information: six tables, detailing quality control for carbohydrate analysis, campaign averages of WIBS-4a data, offline sample timings, cold pool characteristics of case studies, correlations of offline INPS with WIBS particles and mannitol, and correlation of online INPs with WIBS data. It also contains six figures, including a timeseries of all chemical tracers during BACS-I, a timeseries of all chemical tracers during BACS-II, diurnal plots of fluorescent particles and meteorology for BACS-I, diurnal plots for BACS-II, microsocpy images of fungal spores during cold pools/rain, and compiled INP spectra across four IOPs. See DOI: https://doi.org/10.1039/d5ea00129c.
