# Supplementary material for: Convective storms alter bioaerosol populations through cold pools and precipitation
Source: Environ Sci Atmos. 2026 Jan 6;6(3):286–309. doi: 10.1039/d5ea00129c (PMC12863125; doi:10.1039/d5ea00129c)
Supplement: EA-006-D5EA00129C-s001 [file EA-006-D5EA00129C-s001.pdf]

Supplementary Information (SI) for Environmental Science: Atmospheres.
This journal is © The Royal Society of Chemistry 2026

Supplemental Information for

Convective storms alter bioaerosol populations through cold pools and precipitation

Teresa K. Feldman[1], Chamari B. A. Mampage[1], Nicholas M. Falk[2], Janeshta C. Fernando[1], Brian Heffernan[2], Thomas C. J. Hill[2], Drew Juergensen[2], Claudia Mignani[2,3], Marina Nieto-Caballero[2], Leah D. Grant[2], Susan C. van den Heever[2], Paul J. DeMott[2], Sonia M. Kreidenweis[2], Russell J. Perkins[2], Elizabeth A. Stone[1,4]

[1]Department of Chemistry, University of Iowa, Iowa City, IA 52242

[2]Department of Atmospheric Science, Colorado State University, Fort Collins, CO 80526

[3]Water and Soil Resource Research, Institute of Geography, University of Augsburg, 86159 Augsburg, Germany

[4]Department of Chemical and Biochemical Engineering, University of Iowa, Iowa City, IA 52242

Table S1. Limits of detection (LOD) and average spike recovery plus or minus one standard deviation for BACS-I and -II samples.

	**BACS-I**		**BACS-II**	
**Carbohydrates**	**LOD (µg L^{-1})**	**Spike recovery (%)**	**LOD (µg L^{-1})**	**Spike recovery (%)**
Mannitol	1.8	104 ± 5	1.4	105 ± 3
Glucose	1.6	105 ± 5	3.1	105 ± 4
Sucrose	2.2	108 ± 4	3.5	103 ± 5
Fructose	1.7	106 ± 7	3.5	105 ± 5

Table S2. Overview of particles ≥0.8 µm measured with the WIBS-4a at the Semi-Arid Grassland Research Center.[†]

	**BACS-I** (May 23 – June 17, 2022)	**BACS-II** (May 22 – June 23, 2023)
**WIBS-4a particle types**	**Avg.**	**Avg.**
Fluorescent (% total)	19.7	3.6
A (% FP)	5.2	37.2
B (% FP)	54.4	28.1
C (% FP)	2.9	1.7
AB (% FP)	7.4	31
AC (% FP)	0.1	0.04
BC (% FP)	17.5	0.6
ABC (% FP)	12.5	1.3

[†]Sampled at ~1.5 m above ground and roughly 2.7 km from the WIBS-5 sampling site for BACS-I and BACS-II. Similar to the WIBS-5, the WIBS-4a operated at a sample flow rate of 0.3 L min^{-1}, particle sizes were calculated using Mie theory, and fluorescence thresholds were defined as the mean forced trigger signal plus three standard deviations.

Table S3. Timings for samples collected with high flow impactors on selected intensive operation periods (IOPs).

IOP #	*Pre-Rain* Start date and time	End date and time	Duration (h)	*Rain/post-rain* Start date and time	End date and time	Duration (h)
03	5/29/2022 8:23	5/29/2022 14:07	5.7	5/29/2022 14:07	5/30/2022 10:57	20.8
07	6/3/2022 8:21	6/4/2022 8:22	24.0	6/4/2022 8:22	6/5/2022 8:18	23.9
22	6/8/2023 8:37	6/8/2023 15:00	6.4	6/8/2023 15:00	6/9/2023 11:34	20.6
23	6/10/2023 8:40	6/10/2023 13:37	5.0	6/10/2023 13:37	6/11/2023 12:16	22.7
24	6/12/2023 8:25	6/12/2023 15:35	7.2	6/12/2023 15:35	6/13/2023 12:11	20.6

Table S4. Cold pool and precipitation characteristics for selected case studies of discussion.[†]

IOP	Date	Start time	Event	Rainfall (mm)	Wind Direction	U ($m\ s^{-1}$)	ΔU ($m\ s^{-1}$)	θ_v (K)	$\Delta\theta_v$ (K)	RH (%)	ΔRH (%)	Pre-CP [FP] (cm^{-3})	Peak [FP] (cm^{-3})
03	May 29, 2022	13:56	CP	--	W	19.2	6.4	301.8	-10.2	66	39.1	0.23	0.52
		15:31	CP	--	NW	15.1	3.6	303.9	-0.2	59	3.8	0.16	0.21
		20:00	Rain	2.0	--	--	--	--	--	--	--	0.13	0.54
07	Jun 04, 2022	12:28	CP	--	WSW	7.8	4.0	316.4	-2.5	16	-2.1	0.14	0.45
		13:06	CP	--	E	16.3	12.5	315.4	-1.5	19	1.0	0.35	0.43
		13:46	CP	--	NNE	7.9	5.3	316.7	-2.0	20	1.7	0.19	0.20
		14:30	Rain	1.3	--	--	--	--	--	--	--	0.12	0.41
		15:47	CP	--	W	24.1	17.0	313.1	-2.2	20	-5.6	0.11	0.17
		18:12	CP	--	NE	10.0	8.8	313.4	-4.1	35	18.0	0.15	0.19
22	Jun 8, 2023	15:40	CP	--	W	9.0	5.0	309.0	-3.8	80	-4.2	0.03	0.22
		16:05	Rain	2.8	--	--	--	--	--	--	--	0.03	0.59
		16:37	CP	--	N	5.8	5.0	308.7	-0.2	77	6.5	0.11	0.22
23	Jun 10, 2023	13:34	CP/Rain	3.5	NNW	15.2	14.2	304.9	-7.6	75	26.8	0.05	0.37
		15:27	CP/Rain	3.8	WSW	13.9	10.1	303.0	-6.7	80	13.7	0.08	0.77
24	Jun 12, 2023	15:34	CP	--	SE	8.5	4.4	301.4	-4.0	88	7.0	0.11	0.22
		15:45	Rain	5.0	--	--	--	--	--	--	--	0.11	0.32
		16:28	CP	--	NW	8.0	5.5	301.8	-0.1	89	-1.5	0.17	0.29

[†]For meteorological variables, Δ represents the difference between peak values during the cold pool (CP) passage and a ten-minute averaged background, where U is peak wind speed, RH is peak relative humidity, and θ_v is the minimum virtual potential temperature. For fluorescent particles (FP), peak [FP] represents the maximum concentration reached during CP passage, and pre-CP [FP] represents a ten-minute averaged background.

Table S5. Correlation between concentrations of fluorescent particle types and heat-labile INPs at -10, -15, -20, and -25°C for 1-2.5 and 2.5-10 µm size ranges.[†]

INP temp (°C)	Total	FP	A	B	C	AB	BC	ABC	A+AB	Mannitol
						**2.5 – 10 µm**				
-25[a]	0.44	0.31	0.68	**0.74**	-0.13	0.64	-0.30[b]	-0.15	0.67	0.64[a]
-20	-0.07	-0.09	0.35	0.28	-0.36	0.58	-0.47[a]	-0.39	0.53	**0.90***[a]
-15	-0.03	-0.06	0.37	0.32	-0.36	0.60	-0.46[a]	-0.39	0.56	**0.91***[a]
-10[b]	-0.31	-0.50	-0.09	-0.02	-0.46	0.13	-0.47[b]	-0.51	0.07	0.32[c]
						**1 – 2.5 µm**				
-25[b]	0.06	-0.03	0.50	0.14	-0.29	0.49	-0.27	-0.38[b]	0.50	NA
-20	0.20	-0.02	**0.79**	**0.70**	-0.65	**0.84***	**-0.76**	**-0.82[b]**	**0.80***	NA
-15	0.17	0.09	**0.67**	**0.75**	-0.52	**0.72**	-0.64	-0.69[b]	0.69	NA
-10	0.48	0.34	0.48	**0.77**	-0.19	0.44	-0.35	-0.48[b]	0.47	NA

[†]Values reported are Pearson's correlation coefficients (r) for a sample size of nine unless otherwise specified. Fluorescent particle types were averaged for the same duration as the collected sample analyzed for INPs. Samples for correlation analysis included pre-rain and rain/post-rain samples from BACS-I and BACS-II. Correlations with mannitol from 1-2.5 µm and with AC-types were not included due to low detection frequencies. Zeroes were included in averaging periods of WIBS data. Bolded coefficients are for p < 0.05, and bolded coefficients with asterisks are for p < 0.01.
[a] Sample size (n) of eight; [b] Sample size (n) of seven; [c] Sample size (n) of six.

Table S6. Correlation of WIBS particle concentrations with CFDC INP concentrations for IOPs 22, 23, and 24 (8-12 June, 2023, BACS-II).[†]

Particle type	June 8 IOP22	June 10 IOP23	June 12 IOP24
Total	**0.87***	**0.79***	**0.39***
FP	**0.82***	**0.82***	**0.41***
A	**0.57***	**0.62***	**0.67***
B	**0.82***	**0.76***	0.04
C	**0.49***	**0.42***	-0.10
AB	**0.54***	**0.46***	0.01
BC	**0.54***	**0.60***	12

[†]Values reported are Pearson's correlation coefficients (r) for IOP22 (n = 55), IOP23 (n = 37), and IOP24 (n = 70). Correlation coefficients were determined for periods where INP concentrations were greater than the sum of the average background with ten times the standard deviation. AC- and ABC-types were not included due to low detection frequencies. Bolded coefficients are for p < 0.05, and bolded coefficients with asterisks are for p < 0.01.

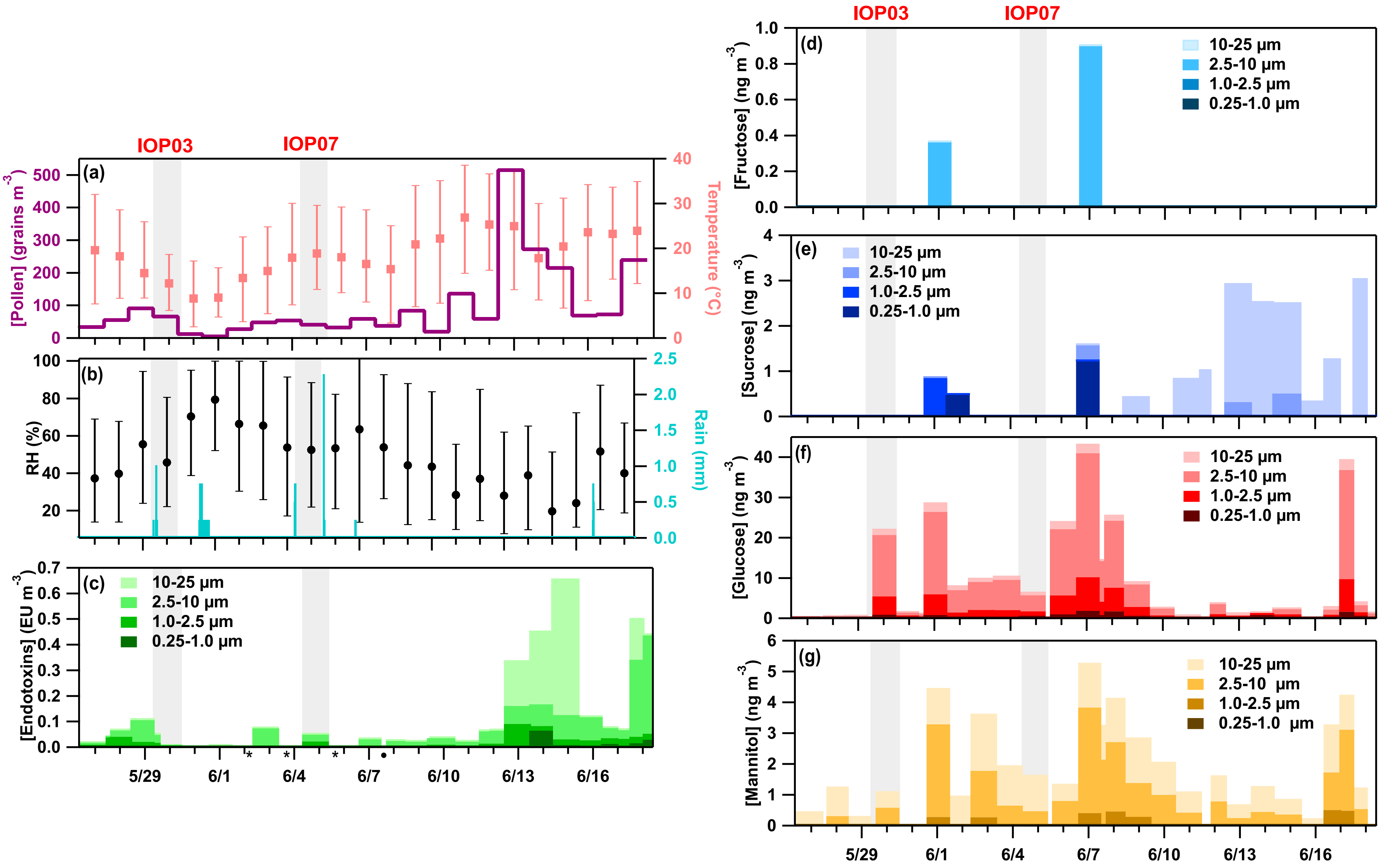

Figure S1. Meteorological data, and chemical and biological tracers for the BACS-I campaign. (a) Plot of the average daily pollen concentration (dark pink) and average daily temperature, with maximum and minimum temperatures (red); (b) plot of the average daily RH, with maximum and minimum values (black), and amount of rainfall (teal) from the National Atmospheric Deposition Program. Analysis of high flow impactors for: (c) endotoxin, (d) fructose, (e) sucrose, (f) glucose, and (g) mannitol. Endotoxin was only analyzed in the 10-25 µm range for select samples based on days with likely dust influence for BACS-I. Of the four samples analyzed for BACS-I, endotoxin was detected in all samples. *1-2.5 µm stage sample not analyzed; · 2.5-10 µm stage sample not analyzed.

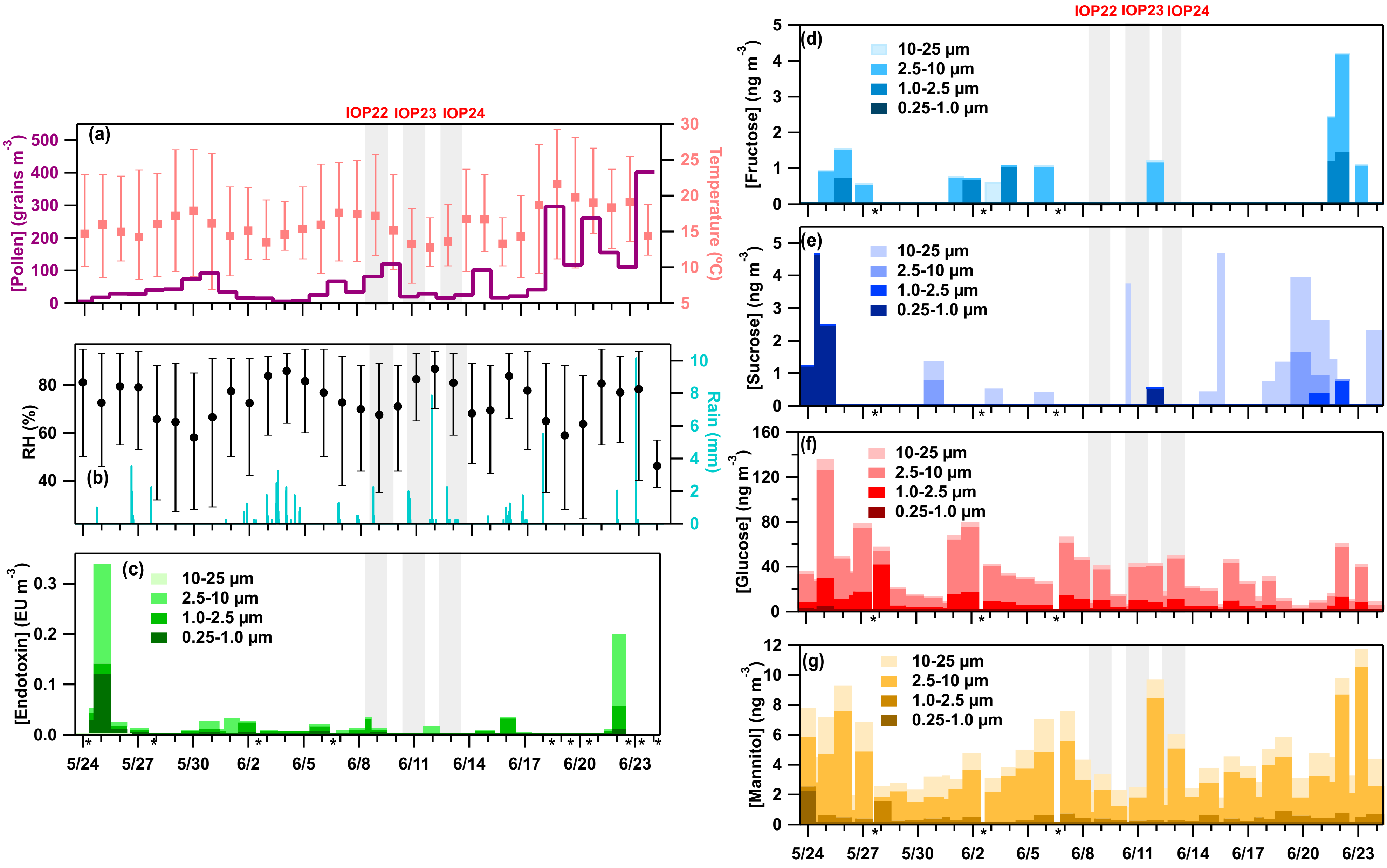

Figure S2. Meteorological data, and chemical and biological tracers for the BACS-II campaign. (a) Plot of the average daily pollen concentration (dark pink) and average daily temperature, with maximum and minimum temperatures (red); (b) plot of the average daily RH, with maximum and minimum values (black), and amount of rainfall (teal) from the soil plot meteorological station summed over 15-minute intervals. Analysis of high flow impactors for: (c) endotoxin, (d) fructose, (e) sucrose, (f) glucose, and (g) mannitol. Endotoxin in particles from 10-25 µm was only analyzed for six select samples from IOPs of interest, but was not detected in any of the samples. *Sample not analyzed.

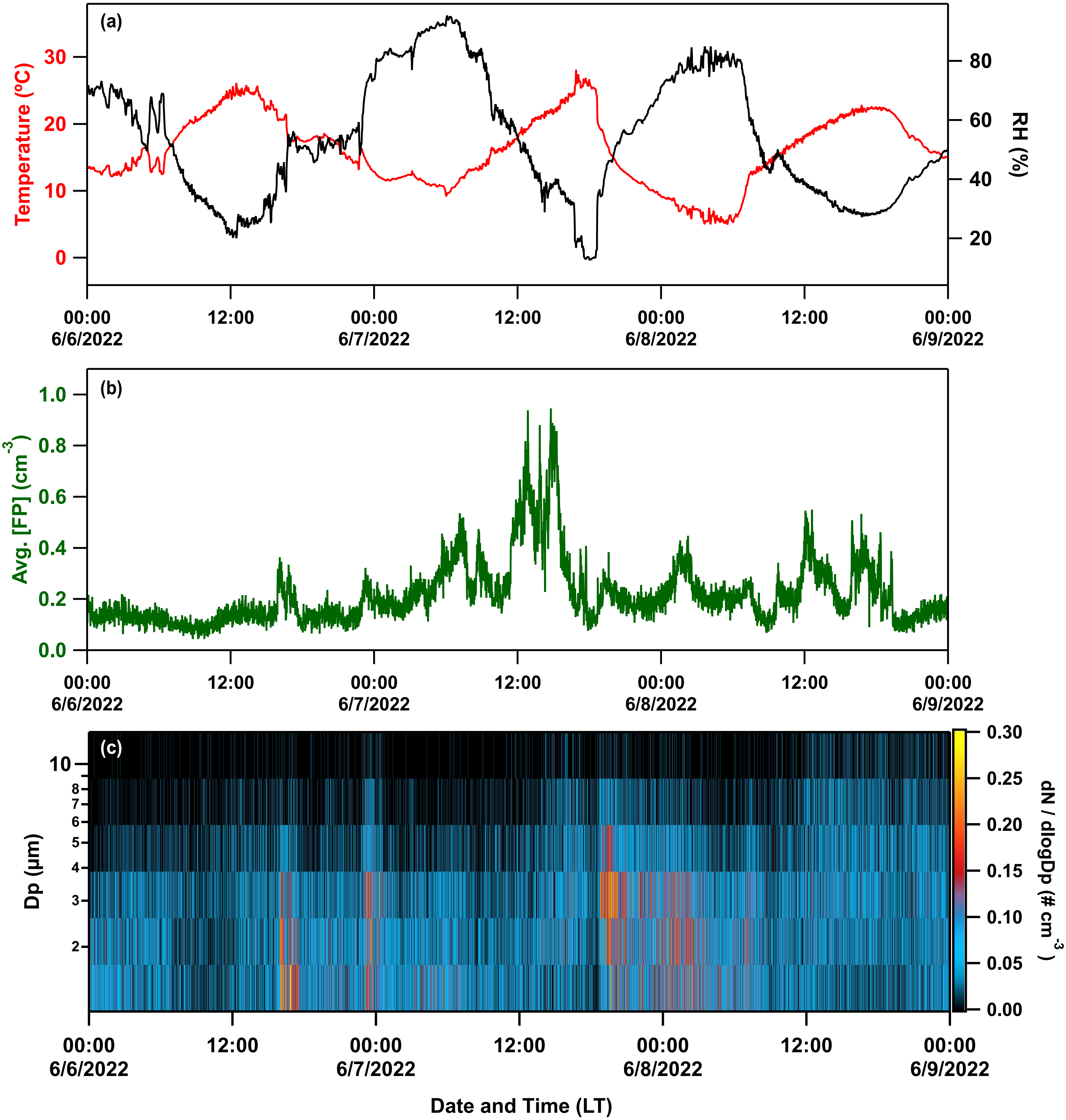

Figure S3. Timeseries of WIBS-5 fluorescent particles showing a diurnal series of fluorescent particles during BACS-I from June 6 – June 9, 2022. (a) Temperature (red) and RH (black) traces; (b) Average (1-min) fluorescent particle concentrations (cm^{-3}) (green) and rainfall amount (teal); (c) Fluorescent particle size distribution from 1.1-13.2 µm.

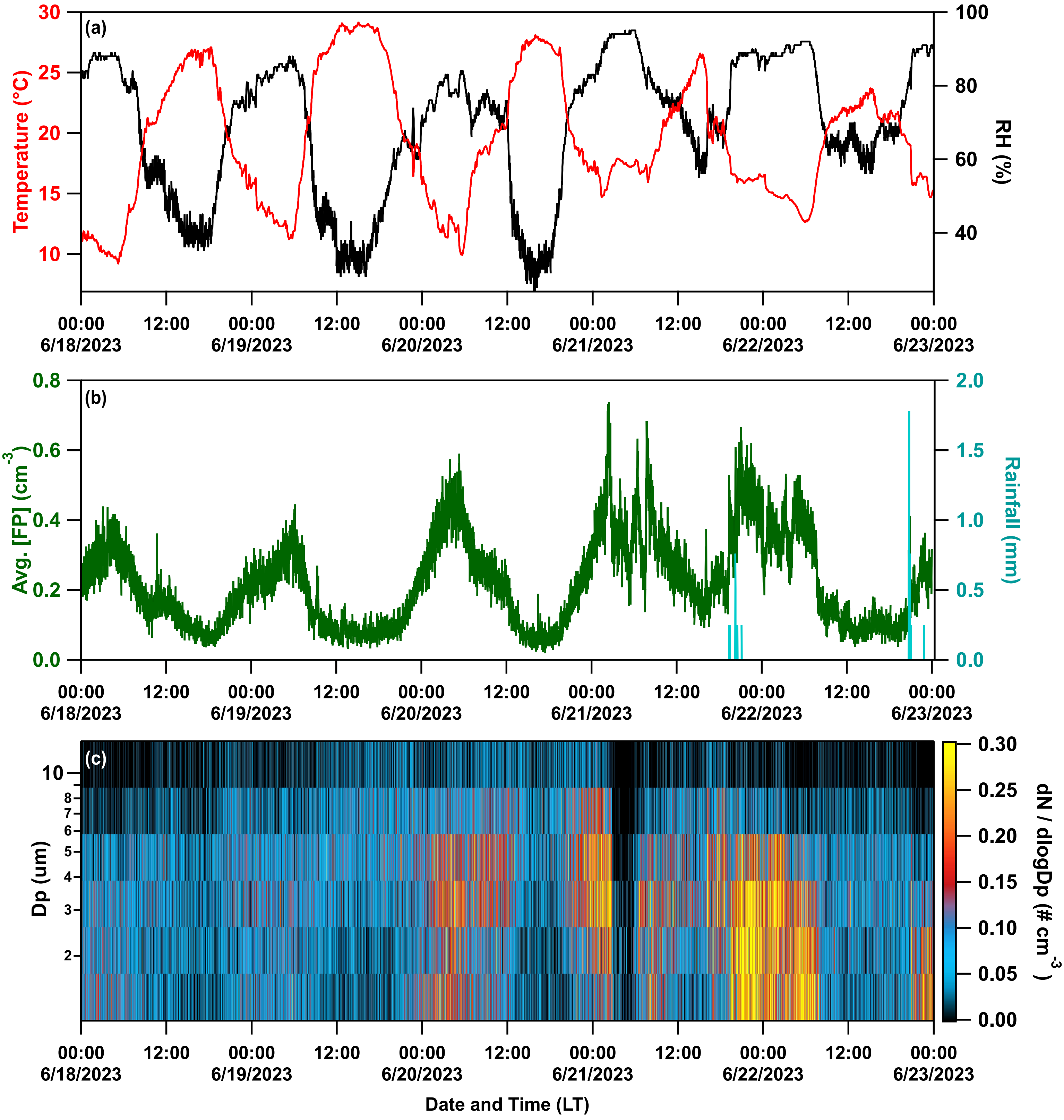

Figure S4. Timeseries of WIBS-5 fluorescent particles showing a diurnal series of fluorescent particles during BACS-II from June 18 – June 23, 2023. (a) Temperature (red) and RH (black) traces; (b) Average (1-min) fluorescent particle concentrations (cm^{-3}) (green) and rainfall amount (teal); (c) Fluorescent particle size distribution from 1.1-13.2 µm.

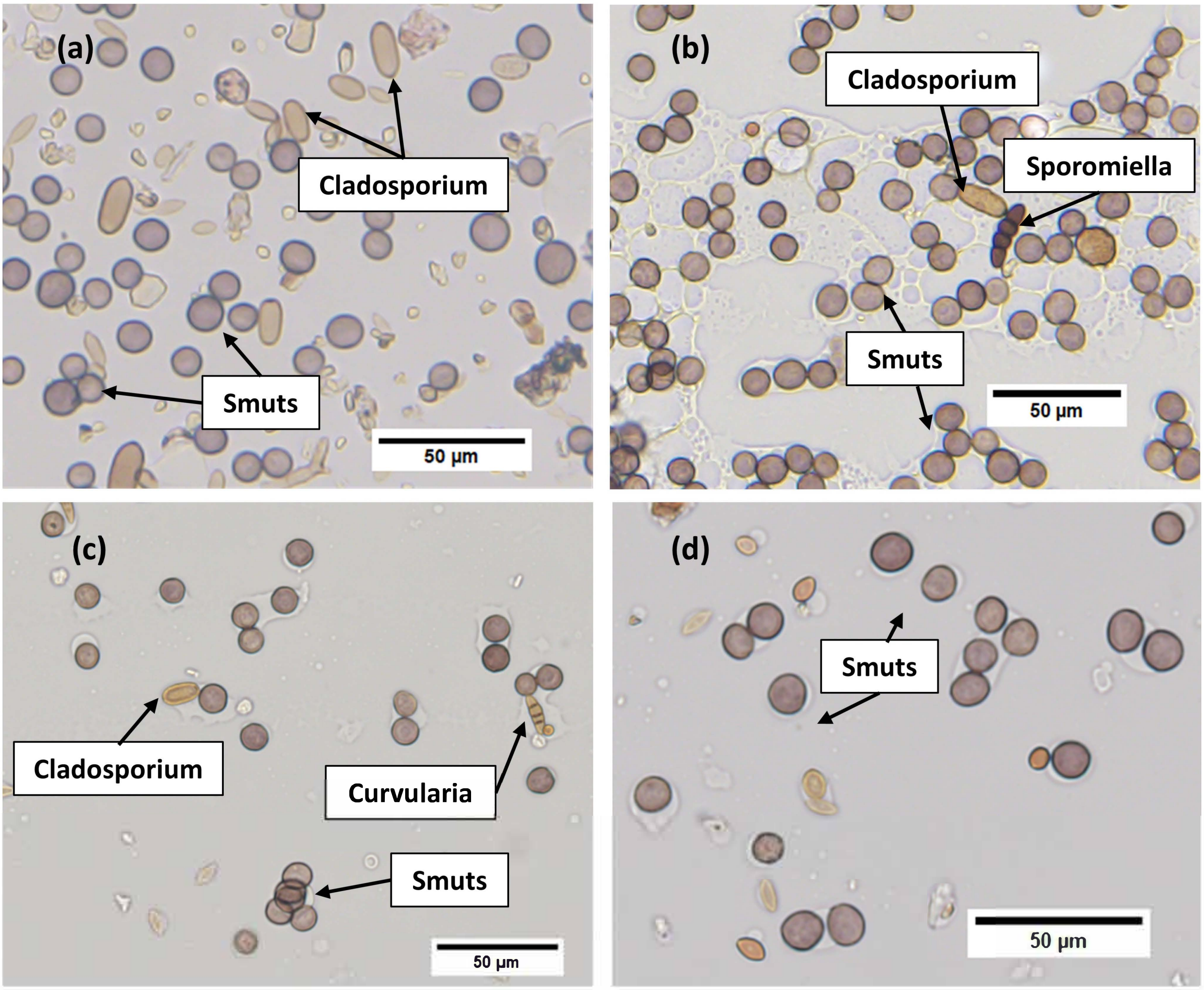

Figure S5. Snapshot of enhanced fungal spore loading during first cold pool and precipitation periods for case studies. (a) June 8, 2023 (15:24-16:24); (b) June 10, 2023 (12:49-13:49); (c) June 12, 2023 (15:37-16:37); (d) June 15, 2023 (14:29-15:29).

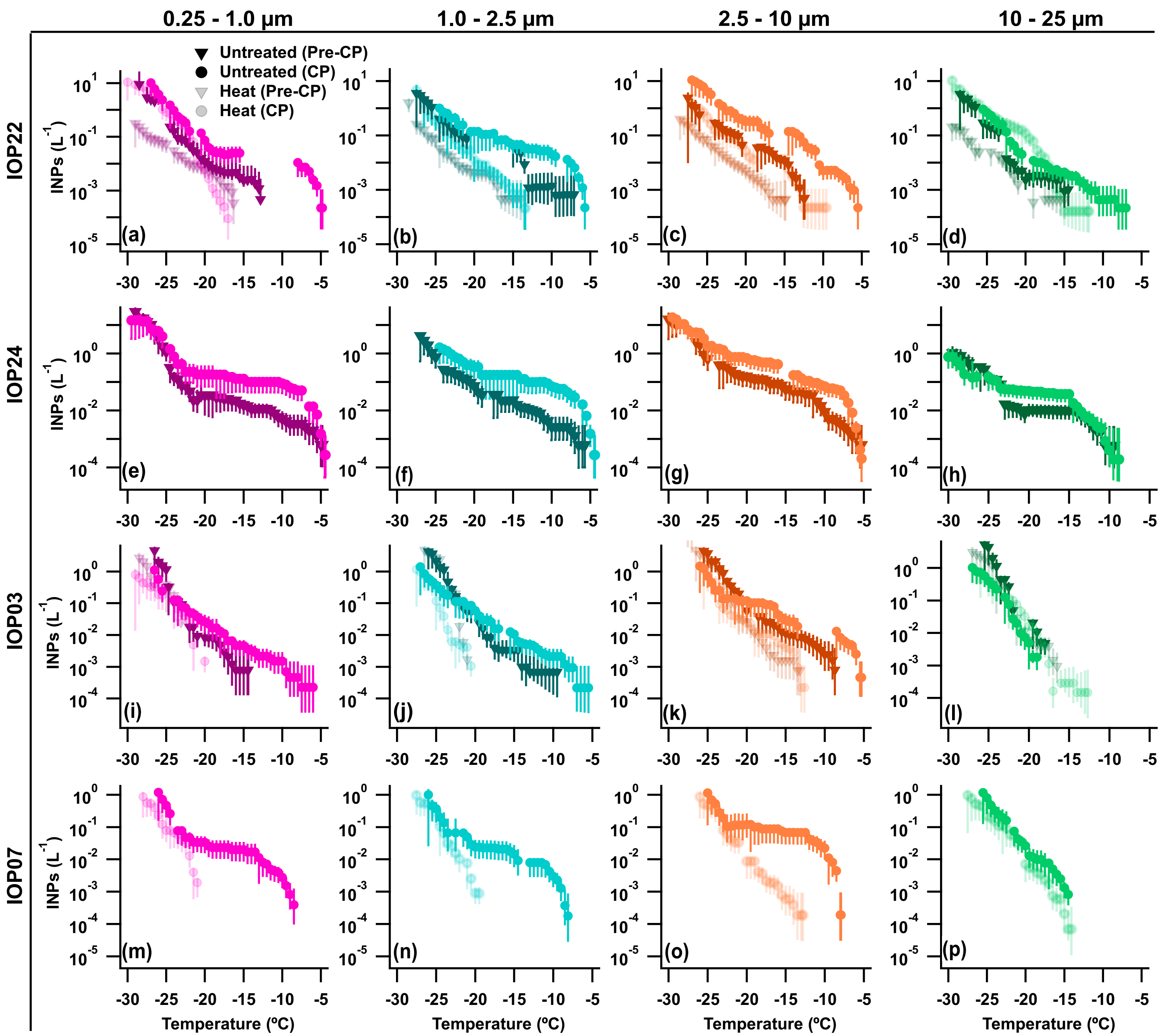

Figure S6. Ice Spectrometer INP analysis for high flow impactor samples taken before the cold pool and precipitation (Pre-CP) (inverted triangles) and collected during the cold pool and precipitation (CP) (circles). Sample timings can be found in Table S3. INPs for untreated (filled) and heat-treated (transparent) samples across four stages are included for (a-d) IOP22, (e-h) IOP24, (i-l) IOP03, (m-p) and IOP07.
